# DSF inactivator RpfB homologous FadD upregulated in *Bradyrhizobium japonicum* under iron limiting conditions

**DOI:** 10.1038/s41598-023-35487-9

**Published:** 2023-05-29

**Authors:** Kunal Dutta, Sergey Shityakov, Fumito Maruyama

**Affiliations:** 1 Laboratory of Chemoinformatics, Infochemistry Scientific Center, ITMO University, Saint Petersburg, Russian Federation; 2 Microbial Genomics and Ecology, The IDEC Institute, Hiroshima University, Higashihiroshima, Japan

**Keywords:** Bacterial genomics, Bacterial systems biology

## Abstract

Phytopathogenic bacteria *Xanthomonas campestris* pv. *campestris* (*Xcc*) causes black rot and other plant diseases. *Xcc* senses diffusible signal factor (DSF) as a quorum-sensing (QS) signal that mediates mainly iron uptake and virulence. RpfB deactivates DSF in this DSF–QS circuit. We examined differential gene expression profiles of *Bradyrhizobium japonicum* under low versus high iron conditions and found that *fadD* and *irr* were upregulated under low iron (log2 fold change 0.825 and 1.716, respectively). In addition to having similar protein folding patterns and functional domain similarities, FadD shared 58% sequence similarity with RpfB of *Xcc*. The RpfB–DSF and FadD–DSF complexes had SWISSDock molecular docking scores of − 8.88 kcal/mol and − 9.85 kcal/mol, respectively, and the 100 ns molecular dynamics simulation results were in accord with the docking results. However, significant differences were found between the binding energies of FadD–DSF and RpfB–DSF, indicating possible FadD-dependent DSF turnover. The protein–protein interaction network showed that FadD connected indirectly with ABC transporter permease (ABCtp), which was also upregulated (log2 fold change 5.485). We speculate that the low iron condition may be a mimetic environmental stimulus for *fadD* upregulation in *B. japonicum* to deactivate DSF, inhibit iron uptake and virulence of DSF-producing neighbors. This finding provides a new option of using *B. japonicum* or a genetically improved *B. japonicum* as a potential biocontrol agent against *Xcc*, with the added benefit of plant growth-promoting properties.

## Introduction

Iron is the most ubiquitous element in the Earth’s crust after oxygen, silicon, and aluminum. Iron usually exists in the ferrous (Fe^2+^) or ferric (Fe^3+^) oxidation state^1^. Ferric iron has relatively low solubility, although it is the predominant form in Earth’s oxygenated environment, and this poses a challenge for bacteria with aerobic metabolism. At low pH, the more soluble ferrous iron is the most abundant in an anaerobic or microaerobic environment^2^. Iron is essential for the activity of regulatory and metabolic enzymes, which is why it is essential for bacterial physiology. However, iron can be toxic to cells because of its potential to transport electrons at physiological pH. Bacteria have evolved mechanisms to actively gather iron from the environment when iron is scarce and to control the availability of intracellular free iron when iron is abundant^3^. This iron homeostasis is crucial for both virulence and normal cellular processes^1^. Ferric uptake regulator (Fur) is a global transcriptional regulator of iron homeostasis, virulence, and oxidative stress^4^. Fur has been widely researched in *Escherichia coli*^5^ and has been shown to regulate iron-dependent expression of over 90 genes^6^. Fur functions as a positive repressor because when it interacts with iron (its co-repressor) it suppresses transcription and when iron is absent it depresses transcription^6^. Fur is a homodimer DNA-binding protein that binds one ferrous ion per subunit. At low iron concentrations, the DNA binding affinity of apo-Fur (Fur without iron) is approximately 1000 times lower than that of Fur and its activity is depressed, resulting in iron absorption, suppression of RyhB small RNA, decreased iron storage, and decreased iron-protein synthesis^6^. Siderophores are extracellular ferric iron chelating molecules that are synthesized by bacteria for iron acquisition^7^. Bacterial strains that are incapable of siderophores production often utilize siderophores produced by other bacterial strains, which gives them a competitive advantage inter-species competition or iron-limiting conditions^8^. Bacteria have other iron acquisition mechanisms, including heme absorption and a ferrous iron transport system^2,9^. However, *fur* mutant strains were found to lack appropriate iron homeostasis, and a high concentration of intracellular iron was detected as a result of constantly high siderophore production^4^. In host plants, the *fur* mutant strains had diminished oxidative stress resistance and decreased virulence traits^4,10^. These findings highlight the importance of iron and Fur in the pathogenicity of phytopathogenic bacteria.

Rhizobacteria are soil-dwelling α-proteobacteria that can build symbiotic relationships with leguminous plants (e.g., soybean)^11^. In a symbiotic relationship, plant growth-promoting rhizobacteria promote plant development through a variety of mechanisms, including but not limited to nitrogen fixation, phosphate solubilization, phytohormone production, antibiotic production, and quorum-sensing (QS) interference^12^. Most bacteria use QS as a cell-density-dependent communication mechanism^13^. QS is mediated mainly by N-acyl homoserine lactone, and the social life of bacteria is partly dictated by QS. In phytopathogenic bacteria such as *Xanthomonas campestris* pv. *campestris* (*Xcc*), QS is mediated by family members of the diffusible signal factor (DSF), such as DSF, *cis*-2-dodecenoic acid (BDSF), *cis,cis*-11-methyldodeca-2,5-dienoic acid (IDSF), *trans*-2-decenoic acid (SDSF) and resuscitation-promoting factor RpfB regulates DSF turnover^14^. Long-chain fatty acid CoA ligase (FadD) sequences of *Escherichia coli*, *Sinorhizobium meliloti*, and *Agrobacterium tumefaciens* are homologous with the RpfB sequence of *Xcc*^15^. Disruption of QS signals by enzymatic conversion of homoserine lactone or DSF is called quorum quenching, and QQ often improves the competitive fitness of bacterial species^16^. *Bradyrhizobium japonicum* is a rhizobacteria that promotes plant growth^12^. In *B. japonicum* strain LO, iron homeostasis and iron-regulated genes are regulated by iron response regulator (Irr) protein and not restricted to heme biosynthesis^5^. In this work, we analyzed differential gene expression levels in *B. japonicum* strain LO under low and high iron conditions, using microarray expression profiles from the NCBI Gene Expression Omnibus dataset (GEO: GSE4143) and protein–protein interactions using STRING. We found that expression of the *fadD* gene of *B. japonicum* was upregulated under the low iron condition (log2 fold change (FC) 0.825). We also found that the amino acid sequences of *B. japonicum* FadD and *Xcc* RpfB shared 58% identity, and that the binding energy of FadD–DSF was significantly higher than that of RpfB–DSF, indicating potential FadD-dependent DSF turnover. We speculate that the low iron condition may be a mimetic environmental stimulus for *B. japonicum* that upregulates *fadD* to deactivate DSF, inhibit iron uptake, and the virulence of DSF-producing neighbors.

## Results

### Differentially expressed genes in the GEO dataset

**Figure 1:**
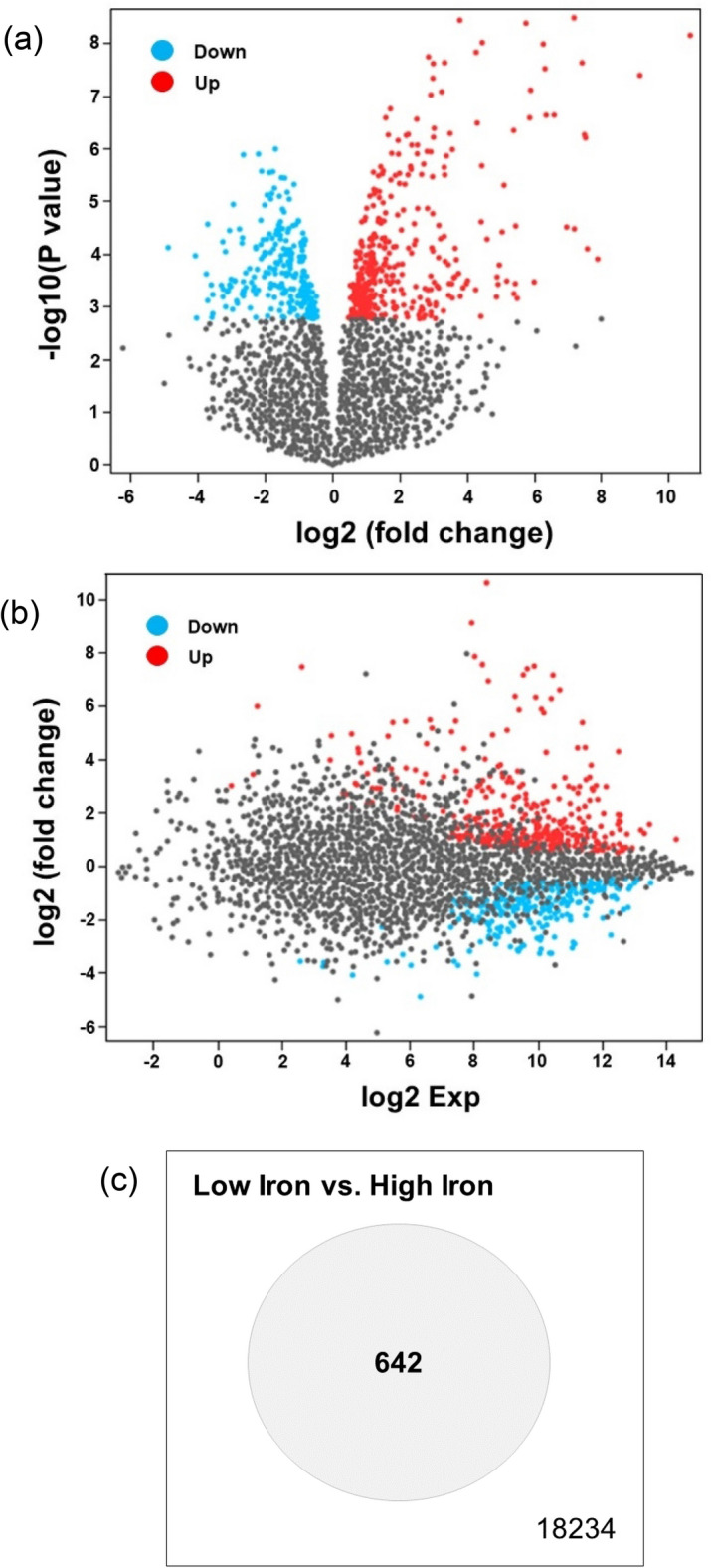
Differentially expressed genes between *Bradyrhizobium japonicum* grown in low and high iron conditions. (**a**) Volcano plot. (**b**) Mean difference plot showing log2 fold change (x-axis) versus average log2 expression values (y-axis). Genes were considered to be significantly differentially expressed when the Benjamini–Hochberg false discovery rate-adjusted *p* value was < 0.05. (**c**) Venn diagram of the overlap in significantly differentially expressed genes between the low and high iron conditions. The Gene Expression Omnibus GSE4143 dataset was used in this analysis. Graphical plots were generated using the GEO2R analysis tool and adapted for better readability.

**Figure 2:**
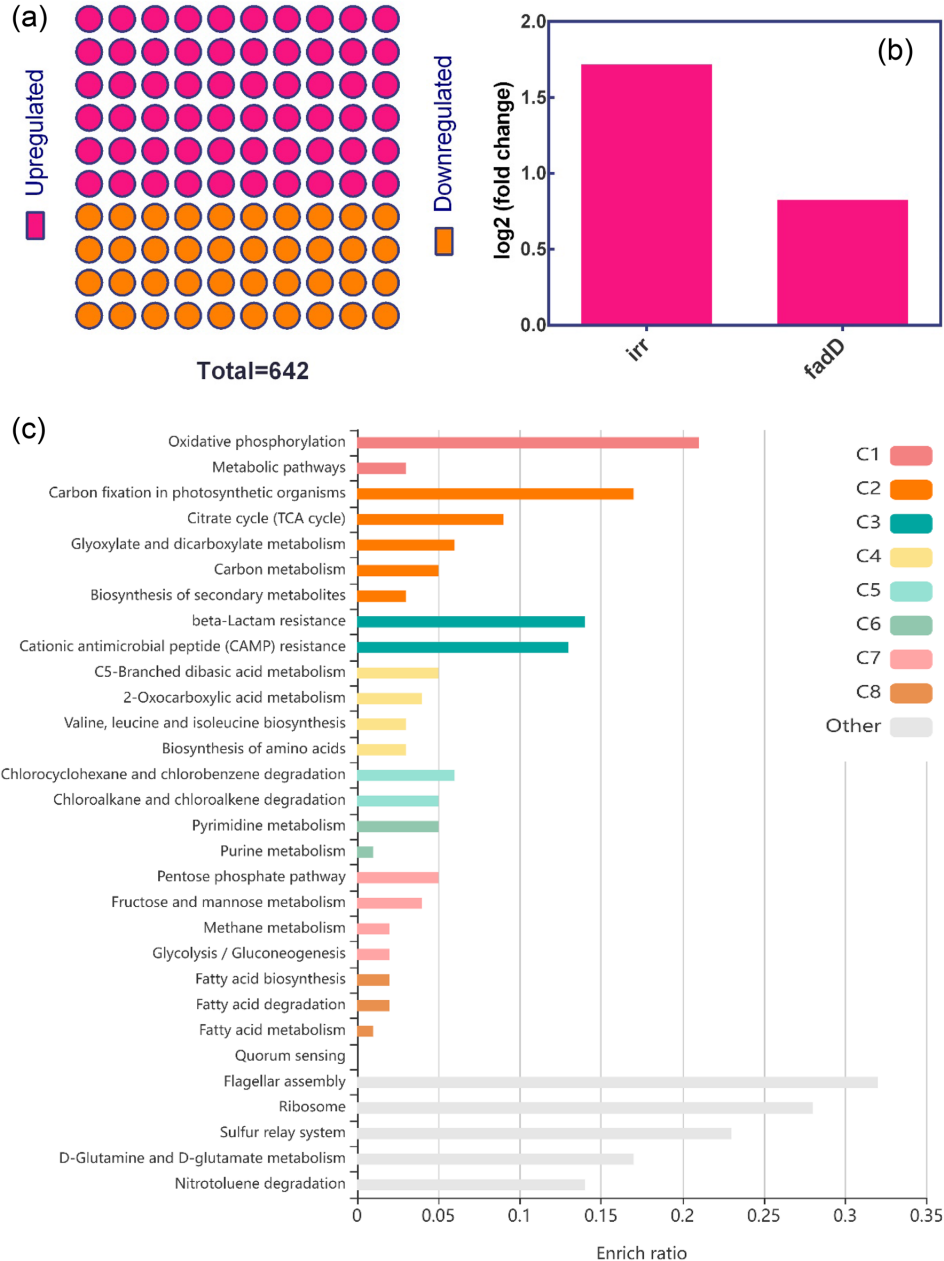
Differentially expressed genes (DEGs) and enriched KEGG metabolic pathways. (**a**) Contingency plot of the 642 DEGs between the low and high iron conditions. (**b**) Differential expression levels of *irr* and *fadD* in low iron compared with high iron conditions. (**c**) KEGG metabolic pathways of the DEGs with no cutoff filter. The quorum sensing pathway had a very low enrichment ratio of 0.01 with one input gene (*fadD*) and 19 background genes. The KEGG pathways were divided into eight main clusters (C1–C8) based on their functions.

Analysis of the GEO dataset GSE4143 showed that 642 genes were significantly differentially expressed (DEGs) under the low iron condition (low versus high iron) (Fig. 1). Only 457 of the 642 DEGs were used for further analyses (Supplementary Table S1). Among the 642 DEGs, 384 were upregulated and 258 were downregulated (Fig. 2a). The *fadD* and *irr* genes of *B. japonicum* were upregulated (log2 FC 0.825 and 1.716, respectively) (Fig. 2b). Notably, the ABC transporter permease gene (*ABCtp, blr3355*) was also upregulated (log2 FC 5.485).

### Gene list functional enrichment analysis

The Kyoto Encyclopedia of Genes and Genomes (KEGG) pathway enrichment analysis with the Benjamini–Hochberg FDR (*P* < 0.05) of the DEGs showed that most of the downregulated genes were involved in flagellar assembly, ribosome, oxidative phosphorylation, metabolic pathways, carbon fixation in photosynthetic organisms, porphyrin and chlorophyll metabolism, carbon metabolism, microbial metabolism in diverse environments, glyoxylate, dicarboxylate metabolism, and biosynthesis of secondary metabolites (Supplementary Table S2), whereas most of the upregulated genes were involved in sulfur relay (*moaE*, *moaD*, *mnmA*) and beta-lactam resistance (*acrB*, *ampC*, *acrA*) pathways (Supplementary Table S3). In addition, we used the KEGG Orthology Based Annotation System (KOBAS) also without the Benjamini–Hochberg FDR filter setting (*p* < 0.05) and identified a QS pathway that had one gene (*fadD*) and 19 background genes (Fig. 2c).

### Protein–protein interaction network

**Figure 3:**
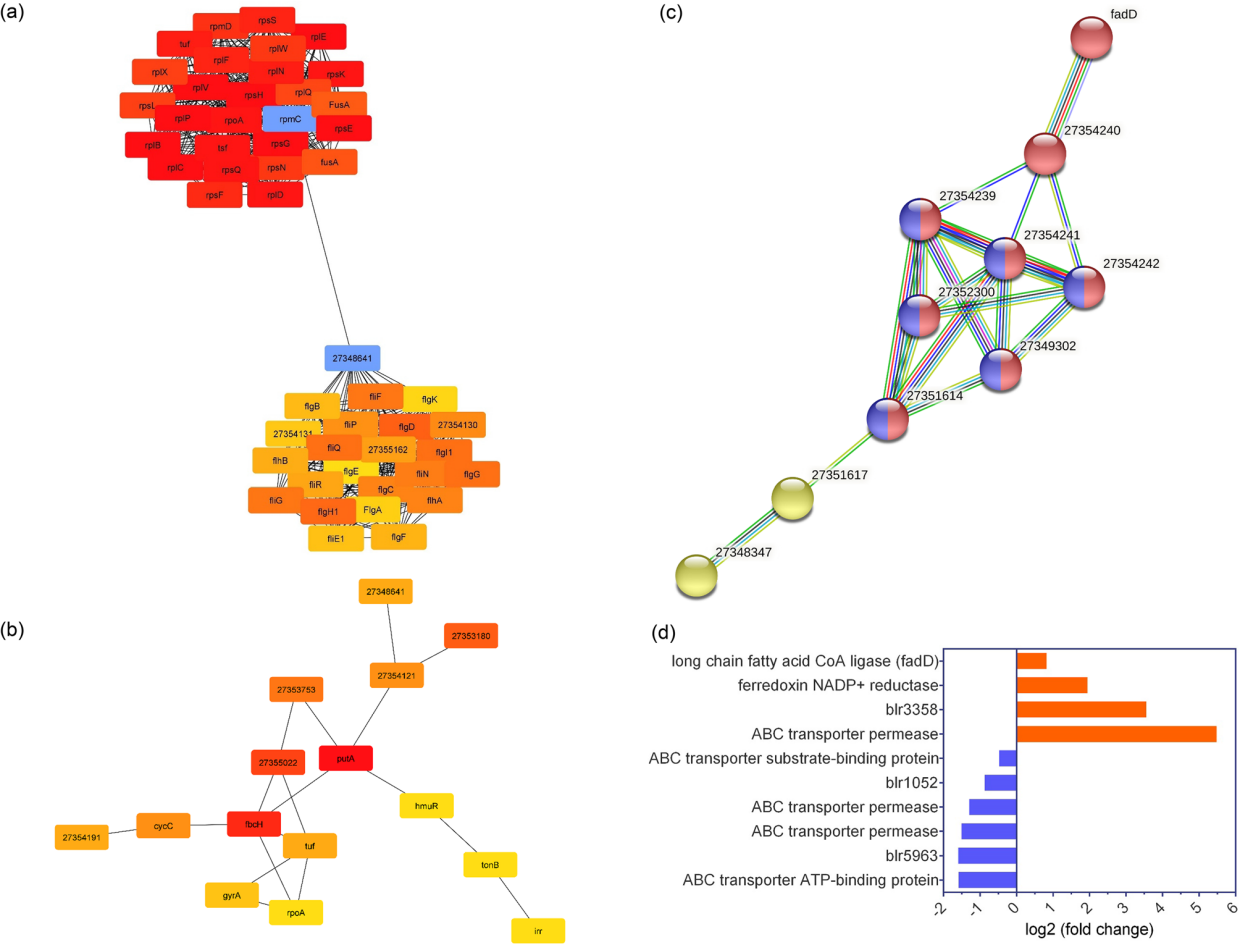
Protein–protein interaction (PPI) network of the differentially expressed genes, and hub and bottleneck nodes. (**a**) Top 50 hub nodes in the PPI network. Connecting hub nodes are in blue boxes. (**b**) Top 15 bottleneck nodes in the PPI network. The Irr protein is bottom right. (**c**) The FadD cluster in the PPI network was separated out and studied using STRING. Nodes are in multiple colors according to the pathways involved: red, quorum sensing (bja02024)^58–60^; blue, ABC transporters (bja02010)^58–60^; mixed, including ferredoxin NADP reductase and bacteria; and yellow, cellular iron ion homeostasis (Local network cluster (STRING), CL: 1531). (**d**) Differential expression levels of node genes involved in various pathways, including quorum sensing and ABC transporter pathways.

We constructed a large protein–protein interaction (PPI) network of the DEGs (Supplementary Fig. S1) and found that the encoded proteins in the top 50 hub nodes were involved in two important KEGG pathways, namely the ribosome and flagellar assembly pathways. The rpmC protein in the ribosome pathway and node 2734684 in the flagellar assembly pathways connect these two metabolic pathways (Fig. 3a). Notably, the top 15 bottleneck nodes included the Irr protein, whereas FadD was not among the top 50 hub or 15 bottleneck nodes (Fig. 3b). The PPI network is composed of subnetworks, including FadD, flagellar assembly, ribosome, and root nodulation clusters (Supplementary Fig. S1). FadD interacts with specific nodes, namely 27354240, 27354241, 27354239, 27354242, 27352300, 27349302, 27351614, 27351617, 27348347 (Fig. 3c). A separate STRING^17^-based analysis of the FadD PPI cluster identified significant QS and ABC transporter pathways with FDR-adjusted *p* values of 7.64e^−09^ and 2.82e^−05^, respectively (Table 1). Furthermore, FadD and 27354240 participated exclusively in the QS pathway, whereas 27354239, 27354241, 27354242, 27352300, 27349302, and 27351614 participated in the QS and ABC transporter pathways. Additionally, nodes 27351614 and 27348347 participated in various pathways, including cellular iron homeostasis (Fig. 3c). The results also showed that FadD was indirectly connected to ABCtp, which was upregulated (log2 FC 5.485) (Fig. 3d).

**Table 1:** KEGG pathways in the FadD cluster from the protein–protein interaction network in Fig. 3c.

Description	KEGG pathway	Count in network	Strength	FDR (*p*-value)
Quorum sensing	bja02024	8 of 273	1.39	7.64e^−09^
ABC transporter	bja02010	6 of 305	1.21	2.82e^−05^

Count in network:

The first number indicates how many proteins in the network are annotated with a particular term. The second number indicates how many proteins in total have this term assigned.

Strength:

Log10(observed/expected). This measure describes how large the enrichment effect is. It’s the ratio between (i) the number of proteins in the network that are annotated with a term and (ii) the number of proteins that expect to be annotated with this term in a random network of the same size.

False Discovery Rate (FDR):

This measure describes how significant the enrichment is. Shown are *p *values corrected for multiple testing within each category using the Benjamini–Hochberg procedure.

### Comparison of FadD and RpfB sequences and structures

**Figure 4:**
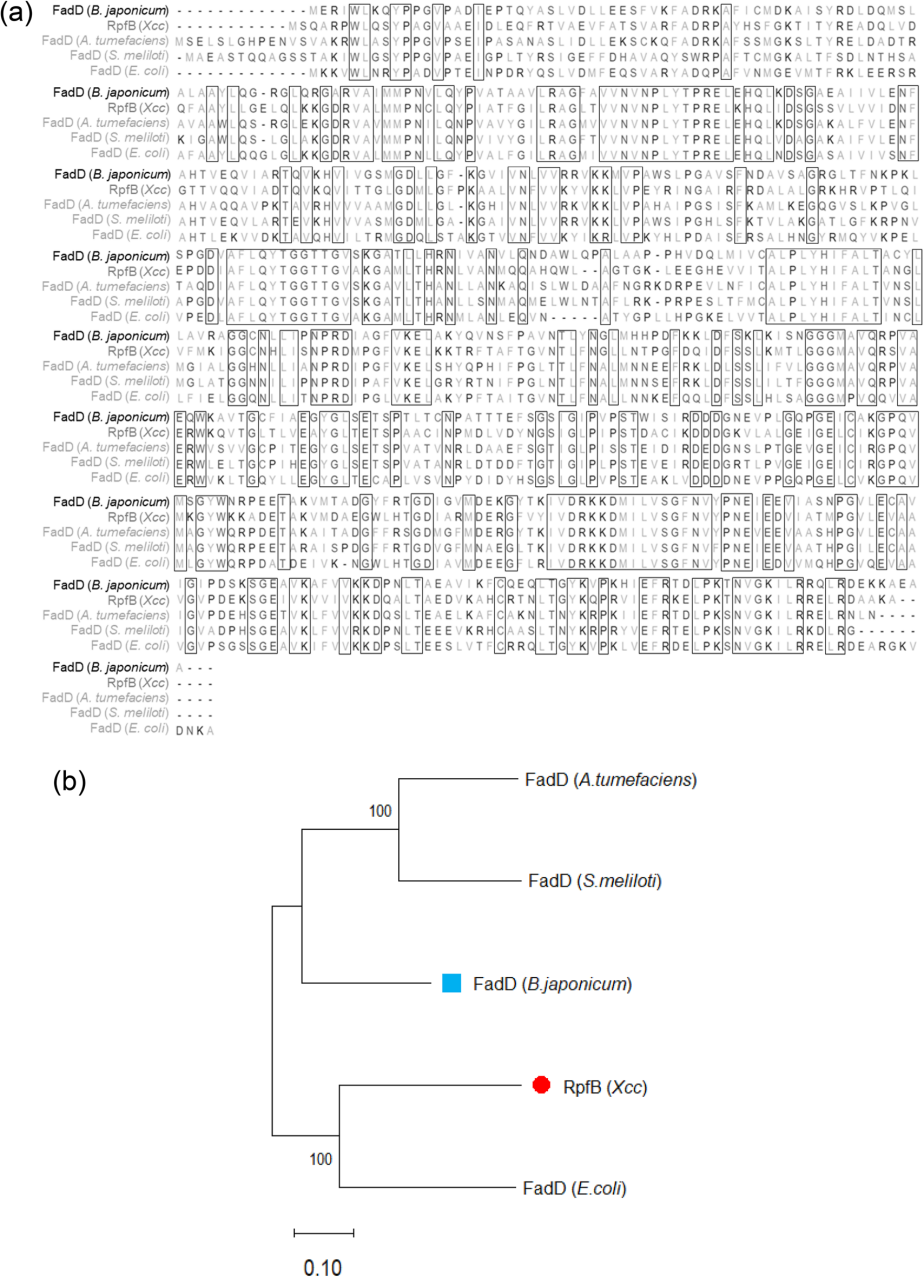
Comparison of the FadD amino acid sequences of *Bradyrhizobium japonicum*, related bacterial species and RpfB of *Xcc*. (**a**) Multiple sequence alignment, and (**b**) phylogenetic tree. The bootstrap values are shown next to the branches.

Alignment of the *B. japonicum* FadD and *Xcc* RpfB amino acid sequences showed that they shared 58% similarity (Supplementary Fig. S2) and had identical protein family domains in very similar positions in their sequences (Supplementary Table S4). A multiple sequence alignment and phylogenetic tree showed that *B. japonicum* FadD significantly resembled other bacterial FadD amino acid sequences already known to be homologous to the *Xcc* RpfB sequence (Fig. 4). Structural assessment of the AlphaFold^18^ predicted protein structures of RpfB (AF-Q8P9K5-F1) and FadD (AF-A0A0A3XRM6-F1) using the RRDistMaps tool in UCSF Chimera^19^ detected similarities in their three-dimensional structures (Supplementary Fig. S2), and *Xcc* RpfB and *B. japonicum* FadD had TM-align^20^ scores of 0.95995 and 0.95828 respectively, confirming the structures were very similar.

### Molecular docking and rescoring

**Figure 5:**
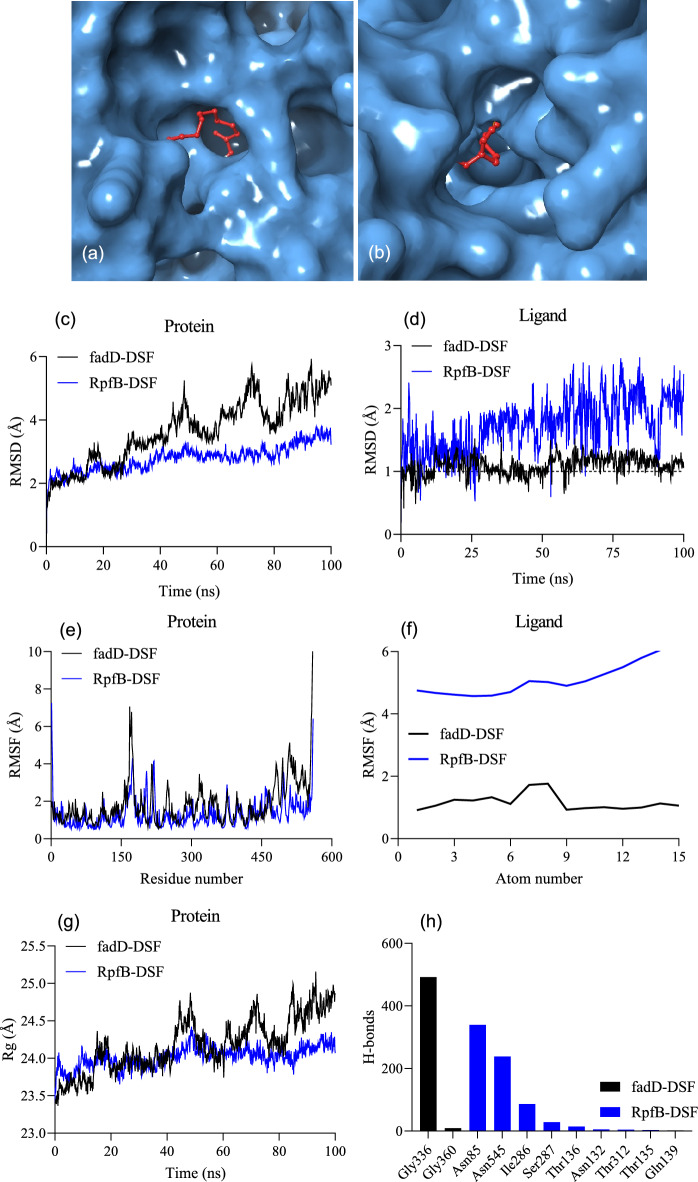
Biophysical interactions between receptors RpfB and FadD and ligand DSF by molecular docking and molecular dynamics (MD) simulations. (**a**,**b**) Molecular docking between DSF and RpfB of *Xcc* (**a**), and between DSF and FadD of *Bradyrhizobium japonicum* (**b**). DSF is indicated by the red ball-and-stick structures. (**c**,**d**) RMSD of the receptors and ligands in the complexes. (**e**,**f**) RMSF of the receptors and ligands in the complexes. (**g**) Radius of gyration (Rg) of the receptor proteins. (**h**) Numbers of H-bonds and the amino acid residues involved in H-bonding calculated using the receptor–ligand complexes generated during the 100-ns molecular dynamics simulations. The threshold is depicted as a dashed line.

Molecular docking scores of RpfB–DSF and FadD–DSF using CB-Dock2^21^ and AutoDock Vina^22^ were not largely different (Fig. 5), whereas the SWISSDock^23^ and fastDRH^24^ scores differed significantly. Specifically, RpfB–DSF had a SWISSDock score of − 8.88 kcal/mol and fastDRH MM-PBSA and MM-GBSA scores of − 22.27 and − 34.07 kcal/mol respectively, whereas FadD–DSF had a SWISSDock score of − 9.85 kcal/mol and fastDRH MM-PBSA and MM-GBSA scores of − 34.02 kcal/mol, and − 38.07 kcal/mol respectively (Table 2)*.* The contact residues between RpfB and DSF were PRO84, ASN85, PRO108, LEU109, ILE130, ASN132, PHE133, LEU154, LEU176, THR259, ALA260, LEU261, PRO262, LEU263, TYR264, ILE286, SER287, ASN288, PRO289, and ARG290, whereas the contact residues between FadD and DSF were ASN236, TRP239, LEU240, PHE265, ALA269, LEU273, ILE331, ASN333, GLY334, GLY335, GLY336, MET337, GLfY358, TYR359, GLY360, LEU361, PRO366, THR367, THR369, and CYS370 (Supplementary Fig. S3). Additionally, the top 10 potential hotspot residues and the top 30 heatmap residues were identified (Supplementary Fig. S4) based on per-residue energy decomposition analysis of multiple docking poses of the ligand^24^. Therefore, they (Supplementary Fig. S4) are maybe pretty useful for future drug–designing endeavors against RpfB *Xcc*.

**Table 2:** Molecular docking energy scores (kcal/mol) for ligand DSF bound to receptors FadD and RpfB and MM-PBSA/GBSA energy terms (kcal/mol) calculated for 100 ns.

Energy terms	FadD-DSF	RpfB-DSF
SWISS-Dock	− 9.85	− 8.88
CB-Dock2	− 5.74	− 5.73
AD-Vina	− 5.65	− 5.52
MM-PBSA^a^	− 27.42	− 22.27
MM-GBSA^a^	− 38.07	− 34.02
ΔE_vdW_	− 42.92	− 28.93
ΔE_el_	− 9.07	− 10.01
ΔG_gas_	− 51.98	− 38.94
ΔG_solv_	45.33^b^/10.93^c^	37.94^b^/15.42^c^
ΔG_pbsa_	− 6.65	− 1.66
ΔG_gbsa_	− 41.06	− 23.52

^a^FastDRH, ^b^MM-PBSA; ^c^MM-GBSA.

### Molecular dynamics simulations

To confirm the molecular docking results, the free energy of binding was estimated based on implicit solvation models for the FadD and RpfB proteins complexed with DSF. The MM-PBSA/GBSA calculations using 100 ns MD trajectories showed that the binding affinity for FadD–DSF was much higher than that for RpfB–DSF (Table 2), which confirmed the molecular docking results. van der Waals forces were the main contributors to the protein–ligand affinity because of the lipophilic nature of the ligand molecule.

To explore the movements of the two complexes during the simulations, root-mean-square deviation (RMSD) and fluctuation (RMSF) values, and the radius of gyration (Rg) were plotted versus time (Fig. 5c–g). The FadD–DSF RMSD values were higher (RMSD = 3.64 Å) than those for RpfB–DSF (RMSD = 2.78 Å), indicating FadD deviated more than RpfB from the initial state (Fig. 5c). The ligand RMSD in the FadD–DSF complex remained unchanged at the 1.0 Å threshold, whereas the ligand RMSD in the RpfB–DSF complex changed greatly probably because DSF was released from the binding site (Fig. 5d). The receptor RMSF values identified amino acid residues associated with the high flexibility of the N- and C-terminal regions of RpfB (Fig. 5e). The RMSF values of DSF showed similar flexibility patterns as the RMSF values for the receptors; that is, DSF was more flexible in the RpfB–DSF complex than it was in the FadD–DSF complex (Fig. 5f). The Rg values showed that RpfB was more compact than FadD in the complexes; that is, Rg values were higher for FadD than they were for RpfB (Fig. 5g).

Numbers of H-bonds between the receptors and their ligands were assessed to determine the contribution of H-bonds to the binding affinity. The binding affinity was high between FadD and DSF although only two amino acids (Gly336 and Gly360) were involved in forming H-bonds. However, nine amino acid residues were involved in forming H-bonds between RpfB and DSF, the binding affinity was low probably because the ligand was released from the binding site (Fig. 5h).

## Discussion

Bacteria are usually surrounded by various strains and species that compete for the limited life resources^25^. Therefore, bacteria have developed strategies to decimate and dislodge their neighbors, including but not limited to secretions to gather resources (e.g., siderophores for iron), injuring or poisoning neighboring cells^26^, and establishing microcolonies while preventing other organisms from doing so^27^. Biofilms are aggregated microcolonies of surface-associated microbial cells enclosed by an extracellular polymeric substance (EPS) and governed mostly by QS^28^. QS also has a crucial role in regulating virulence and biofilm formation in *Xcc*^29^. Conversely, plant growth-promoting rhizobacteria such as *B. japonicum* can promote plant growth by QS interference or quorum quenching of adjacent phytopathogenic bacteria^10,12^. Recently, there has been a major focus on QS signals, including DSF family signals^30^, and QS was found to be associated with advantages or competitive interactions^26^. DSF and BDSF are QS process mediators in *Xcc*^14^, and RpfB can deactivate DSF by β-oxidation via its fatty acyl-CoA ligase activity^31^. Furthermore, DSF may potentially impair the development, growth, and immunity of host plants^31^. In *Xcc*, high extracellular concentrations of DSF activate the RpfC–RpfG system at high cell densities, relieving the inhibition of c-di-GMP on the global transcription factor Clp that regulates the expression of several virulence factors and iron absorption^32^. *Xcc rpfF* produced *DSF* and *rpfF* homolog mutant *Xanthomonas oryzae* pv. *oryzae* (*Xoo*) strains that were deficient in growth and DSF production^33^. *Xoo* is a phytopathogenic bacteria that causes bacterial blight in rice plants. *Xoo rpfF* homolog mutant strains showed unusual tetracycline susceptibility under low iron conditions and tetracycline resistance upon iron supplementation^33^. The iron-dependent pathogenicity of *Xoo* in rice plants has been reported^34^. *Xcc*, can produce xanthoferrin, a siderophore that is necessary for maximum virulence and growth under low iron conditions^35^. Xanthoferrin can bind with ferric iron and enhance growth of *Xcc* in host plants^35^. These findings show the importance of iron and the DSF-dependent QS system for the manifestation of virulence traits of phytopathogenic bacteria.

FadD sequences of *E. coli, S. meliloti,* and *A. tumefaciens* are homologs of the *Xcc* RpfB sequence^15^. However, homology between *B. japonicum* FadD and *Xcc* RpfB has not been reported previously. Alignment of *B. japonicum* FadD and *Xcc* RpfB amino acid sequences showed that they shared 58% similarity (Supplementary Fig. S2). Sequence similarity offers insights into the functions of proteins^36^. TM-align scores of the three-dimensional structures of two model proteins can be obtained by comparing their residue equivalencies^20^. *Xcc* RpfB and *B. japonicum* FadD had TM-align scores of 0.95995 and 0.95828 respectively, which is indicative of similar protein folding patterns. Thus, in addition to sharing sequence similarity, they shared protein folding patterns and protein family domains (Supplementary Table S4), indicating potential functional similarities between the two proteins. Furthermore, the RMSD of RpfB in the RpfB–DSF was lower than the RMSD of FadD in FadD–DSF throughout the MD trajectories. RMSD measures the difference between the initial and final structures of a protein. Discrepancies during the MD trajectory of a protein structure may indicate its conformational stability^37^. For FadD, the RMSD fluctuations did not exceed approximately 4–6 Å (approximately 40–100 ns), indicating a small fluctuation range. For the ligand in the two complexes, the RMSD fluctuations showed opposite trends. The RMSD fluctuations for DSF were higher in the RpfB–DSF complex than they were in the FadD–DSF throughout the MD trajectories (Fig. 5). Small RMSD fluctuations are indicative of stable ligand binding^38^. RMSF quantifies the average displacements of amino acid residues from a reference point over an MD trajectory, and therefore RMSF can identify structural regions that deviate most from their initial structure^39^. Notably, some protein regions of RpfB–DSF and FadD–DSF, approximately 150–160 and 455 to the C-terminal end, exhibited similar fluctuations and, on average, the RMSF of RpfB–DSF and FadD–DSF were broadly similar, indicating satisfactory receptor–ligand interactions. The Rg indicates the distribution of atoms around the axis of a protein, and is a measure of the distance between the point while it is spinning and the point where the transfer of energy has the greatest possible impact^40^. For RpfB in RpfB–DSF, the Rg remained between 23.5 Å and 24.5 Å. For FadD in FadD–DSF, the Rg began to fluctuate after 40 ns then stayed close to 25 Å up to 100 ns. The, more than 500 H-bonds that were predicted to formed by GLY336 of FadD–DSF are indicative of the single dominance of GLY336 in the receptor–ligand interactions, whereas numerous amino acid residues were predicted to form H-bonds in RpfB–DSF. Because H-bonds help create and stabilize protein structures^41^, the formation of H-bonds throughout the MD trajectory suggests stable receptor–ligand interactions in RpfB–DSF and FadD–DSF.

Statistically significant differences in gene expression levels between two experimental conditions are used to identify DEGs^42^. We identified 642 DEGs in *B. japonicum* by comparing gene expression levels under low and high iron conditions (Fig. 1). Irr protein and FadD are important for iron homeostasis and QS or quorum quenching. We found that *fadD* and *irr* of *B. japonicum* were upregulated under the low iron condition (log2 FC 0.825 and 1.716, respectively). Furthermore, report indicated that Irr protein was required for an adequate sense of cellular iron status^5^. And, absence of a normal iron response and iron-associated genes in an *irr* mutant strain was found to decrease the total cellular iron content^5^. These characteristics of the Irr protein explain the upregulation of *irr* under the low iron condition. However, the significance of *fadD* upregulation in *B. japonicum* under low iron conditions has not been explained in previous studies.

In the present study, the gene list functional enrichment analysis identified the flagellar assembly and ribosome pathways as the top two pathways with FDR-adjusted *p* values < 0.05 (Supplementary Table S2), and genes associated with these two metabolic pathways were among the top 50 hub nodes in the PPI network (Fig. 3a). Furthermore, the hub nodes that connect these two pathways, rpmC from the ribosome network and 27348641 from the flagellar assembly network, were found to be critically important (Fig. 3a). However, only Irr protein was among the top 15 bottleneck nodes of the entire PPI network (Fig. 3b), although FadD was not among the top 50 bottleneck/hub nodes. Notably, the expression level of *fadD* (log2 FC 0.825) was almost half that of Irr protein (log2 FC 1.716). This finding raises the question of why high *fadD* expression occurs in *B. japonicum* when iron availability was restricted.

In bacteria, iron plays crucial physiological roles in DNA replication, transcription, metabolism, energy production through respiration, and pathogenicity^3^. Low iron conditions can trigger processes that prevent iron uptake by other microorganisms, thereby ensuring optimal conditions for the survival of self. The ferric iron binding transcription factor XibR, which coordinately controls the expression of genes related to iron metabolism, chemotaxis, and flagellar motility, was found to be necessary for optimal virulence of *Xcc*^43^. In low-iron conditions, *XibR* mutant strains showed decreased growth and decreased intracellular iron concentrations^43^. The significance of iron metabolism in the pathophysiology of bacteria is further supported by the finding that the strong bactericide Xinjunan (dioctyldiethylenetriamine) acted by altering cellular iron metabolism^44^.

In addition to *fadD*, other genes such as *fadE* and *fadR* have been shown to play critical roles in β-oxidation in *E. coli*^45^. Interestingly, *fadE, fadF, fadG, fadH, fadL*, and the transcriptional regulator *fadR* were also found to be involved in bacterial lipid metabolism^46^, but none of these genes were differentially expressed in *B. japonicum* when compared low versus high iron conditions (Supplementary Table S1). During resource-limiting conditions, bacteria generally opt for energy-efficient pathways and nonessential transcription is typically prevented by operons such as the lactose operon in *E. coli*^47^. We found that *fadD* was upregulated in *B. japonicum* under the low iron condition (Fig. 3c), implying that the high expression of *fadD* may be attributable to other factors. The STRING-based analysis of the FadD cluster in the PPI network (Supplementary Fig. S1) found two crucial KEGG pathways: the QS and ABC transporter pathways (Table 1). However, the STRING- and KEGG-based pathway predictions have limitations because these methods are not optimized or adapted for multi-species interactions. Indeed, a homogeneous or isogenic bacterial community is rare because a wide range of microorganisms are typical inhabitants of bacteria^48^. Moreover, bacteria can injure and outcompete their neighbors when resources are scarce through their cell signaling or metabolic pathways^27^. Among the other DEGs, *ABCtp* (blr3355) was upregulated (log2 FC 5.485), and was indirectly connected to FadD (Fig. 3d). Permeases are membrane transporter proteins^36^ that can transport proteins out of cells. The high expression of *ABCtp* (blr3355) under the low iron condition indicates that it was required and probably linked to the extracellular transport of FadD. Furthermore, the ABC transporter was found to be upregulated in *Streptococcus pneumoniae* by two different QS mechanism^49^.

**Figure 6:**
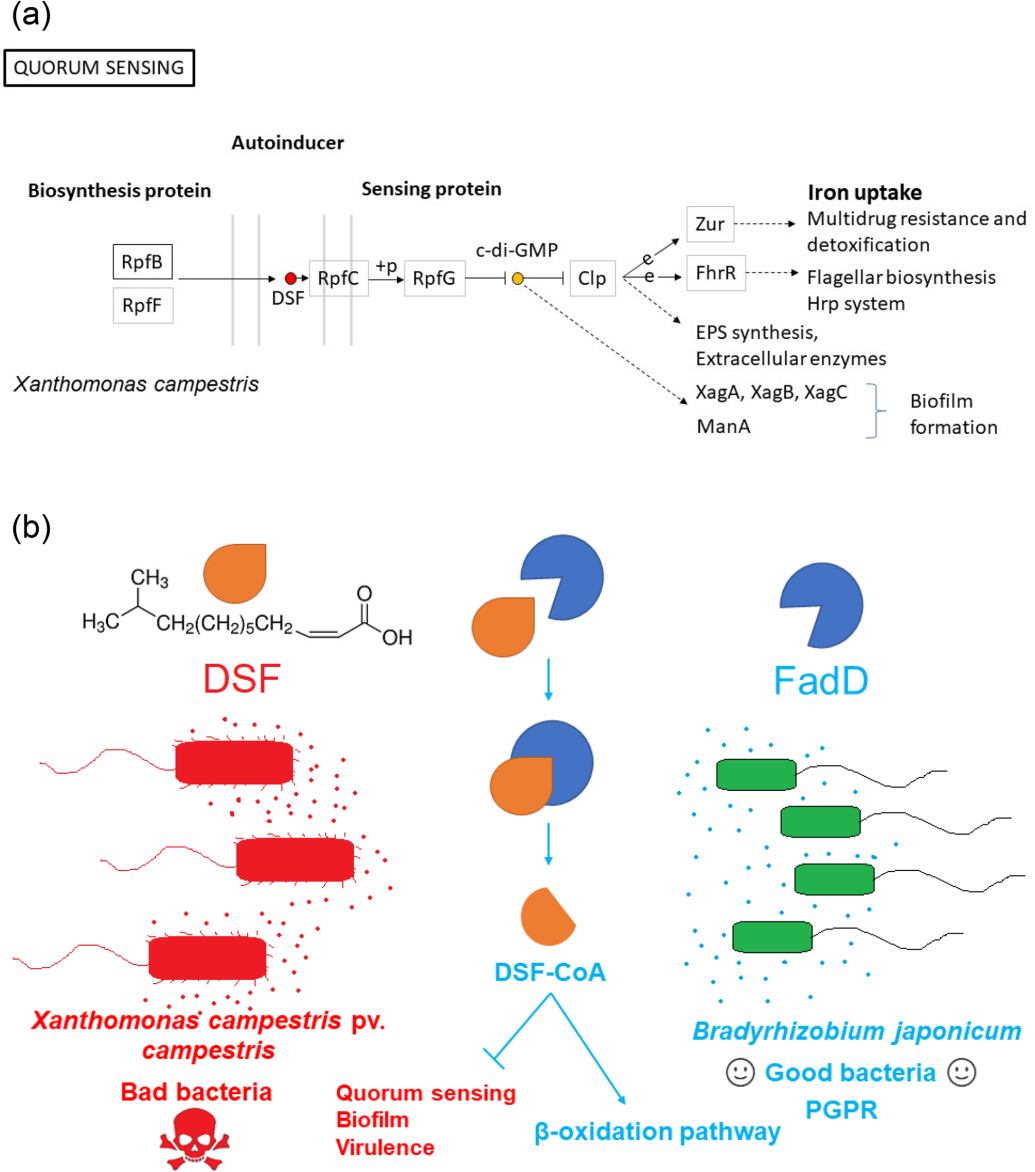
Proposed molecular signaling cascade of DSF-mediated cellular responses. (**a**) DSF-mediated quorum sensing in *Xanthomonas campestris*, obtained from KEGG (bja02024)^58–60^ (**b**) Possible mode of action of inter-species communication via DSF of *Xcc* and the RpfB homolog FadD of *Bradyrhizobium japonicum*.

QS plays an important role in inter-species rivalry^50^ and cooperation^51^, and QS inhibition across species has been reported^52^. RpfB deactivates DSF in *Xcc*^14^, which can disrupt the cyclic di-GMP- and Clp-mediated signaling circuit of cellular iron absorption^31,32,53–56^ similar to *Xanthomonas campestris* (Fig. 6a). Furthermore, *B. japonicum* FadD and *Xcc* RpfB are homologous (Fig. 4) and a significant difference in the binding energies of FadD–DSF and RpfB–DSF was detected (Supplementary Table S3), which indicates potential FadD-dependent DSF turnover (Fig. 6b). Therefore, we speculate that the low iron condition may be a mimetic environmental stimulus for *fadD* upregulation in *B. japonicum* to deactivate DSF and inhibit iron uptake and virulence of DSF-producing neighbors. This may explain why *fadD* and *ABCtp* were upregulated in *B. japonicum* under the low iron condition.

## Conclusions

We analyzed differential gene expression of *B. japonicum* under low and high iron conditions and found that FadD, Irr, and ABCtp genes were upregulated (log2 FC 0.852, 1.724, and 5.485, respectively). The PPI network indicated that FadD was indirectly connected with ABCtp. We identified similar protein folding patterns, functional domains, and 58% sequence similarity between FadD of *B. japonicum* and RpfB of *Xcc*. We also identified QS and ABC transporter pathways by STRING-based analysis of the DEGs. Molecular docking showed a significant difference in binding energies between FadD–DSF and RpfB–DSF, indicating a potential FadD-dependent DSF turnover that was supported by the MD simulations. We speculate that low iron conditions may be a mimetic environmental stimulus for *fadD* upregulation in *B. japonicum* to deactivate DSF-mediated iron uptake and the virulence of DSF-producing neighbors. Our results provide a new option of using *B. japonicum* or genetically modified *B. japonicum* as a biocontrol agent against plant diseases caused by *Xcc*. However, to help assure agricultural production in the face of climate change, exploratory *FadD* gene editing research may be an important focus for future studies.

## Methods

### Microarray data and literature search

A literature search was performed with keyword combinations such as plant growth-promoting rhizobacteria, PGPR, *Bradyrhizobium japonicum*, quorum sensing, iron, and nodulation using Google Scholar (https://scholar.google.com/) and PubMed (https://pubmed.ncbi.nlm.nih.gov/). We also searched the microarray datasets in the NCBI Gene Expression Omnibus (GEO; https://www.ncbi.nlm.nih.gov/geo/) and selected the GSE4143 dataset for analysis (Table 3). GEO samples GSM94778, GSM94780, and GSM94781 were grown in low iron conditions, and GEO samples GSM94783, GSM94784, and GSM94785 were grown in high iron conditions. Briefly, *B. japonicum* LO strain was grown in a modified GSY medium with no exogenous iron source to yield 0.3 µM iron concentration in the medium (low iron condition), and in a modified GSY medium supplemented with 12 µM FeCl_3_.6H_2_O (high iron condition)^5^.

**Table 3:** Details of the NCBI Gene Expression Omnibus dataset used in this study.

Datasets	Sample platform	Samples	Platform organism	Sample organism
GSE4143	GPL3401	Low iron	*Bradyrhizobium diazoefficiens USDA 110*	*Bradyrhizobium japonicum* strain LO
		GSM94778 GSM94780 GSM94781		
		High iron		
		GSM94783 GSM94784 GSM94785		
Process	Grown under an iron limitation or under iron sufficiency and compared to each other by whole genome microarray analysis^5^			

### Differential gene expression

Differentially expressed genes (DEGs) between *B. japonicum* grown in the low and high iron conditions were identified using GEO2R (https://www.ncbi.nlm.nih.gov/geo/geo2r/)^57^. Genes within the cutoff criteria of FDR-adjusted *P*-value < 0.05 (Benjamin–Hochberg) were considered DEGs. Log transformation of the data was set to “auto-detect” mode with force normalization and limma precision weights (Vooma). NCBI-generated category of platform annotation was selected to display on results. The results were obtained in tab-delimited format and exported to Microsoft Excel for further analysis.

### Functional enrichment and ontology of DEGs

The gene symbols of the significant DEGs were used for gene set functional enrichment using KOBAS-Kyoto Encyclopedia of Genes and Genomes (KEGG)^58–60^ pathways (http://kobas.cbi.pku.edu.cn/)^61^ with FDR-adjusted *P* value < 0.05 as the cutoff for significance. The results were also saved without an FDR-adjusted *P* value of < 0.05 for comparative analysis.

### PPI network construction and identification of hub and bottleneck nodes

The gene symbols of the DEGs were used to construct the entire PPI network using STRING (https://string-db.org/). The DEGs were annotated with gene ontology terms under the three main categories, biological process, molecular function, and cellular component, as well as local network cluster (STRING), keywords (Uniport), and protein domains and features (InterPro)^62^ with medium confidence settings (i.e., a combined score > 0.4), and saved as short tabular text output. Subsequently, the PPI network topology was exported directly from the STRING website to Cytoscape (v3.9.1)^63^ (http://www.cytoscape.org/) for visualization. The top 50 hub nodes and top 15 bottleneck nodes were identified using the cytoHubba (v0.1) plugin app with the Maximal Clique Centrality (MCC) scoring method^64^. Nodes that interacted with *fadD* were identified and studied separately.

### Comparisons of FadD and RpfB

Protein sequences of FadD of *B. japonicum* (UniProt: A0A0A3XRM6), RpfB of *Xcc* (UniProt: Q8P9K5), FadD of *E. coli* (GenBank: UBF39181.1), *Agrobacterium tumefaciens* (GenBank: CAD0207327.1), *Sinorhizobium meliloti* SM11 (GenBank: AEH77411.1) were aligned using MUSCLE (MEGA v11)^65^. The evolutionary history was inferred using the maximum likelihood method and JTT matrix-based model^66^, and nearest neighbor interchange distance was used as a tree inference option and tested by 1000 bootstrap replications. Evolutionary analyses were conducted in MEGA v11^65^. The protein domains of FadD of *B. japonicum* and RpfB of *Xcc* were annotated using InterProScan^67^. The three-dimensional protein structures were compared using TM-align^20^ and the RRDistMaps tool in UCSF Chimera^19^.

### Molecular docking and rescoring

The AlphaFold^18^ protein structure files of RpfB (AF-Q8P9K5-F1) and FadD (AF-A0A0A3XRM6-F1) were used as the receptors, and the SDF file of diffused signaling factor (DSF), cis-11-methyl-2-dodecenoic acid (PubChem ID: 11469920) was obtained from the PubChem database and used as the ligand. Auto blind docking was performed using CB-Dock2 (https://cadd.labshare.cn/cb-dock2/php/index.php)^21^ with 10 repeats with unaltered parameters, and averages were used for the interpretations. The best-hit ligand–receptor structures were downloaded, and screenshots of the contact residues were saved for further analysis. The receptor–ligand interactions were visualized using free Maestro v13.2 (Maestro, Schrödinger, LLC, New York, NY, 2021)^68^. SWISSDock (http://www.swissdock.ch/docking)^23^ was also used with the same receptor and ligand. We also used fastDRH (http://cadd.zju.edu.cn/fastdrh/overview)^24^ for high-speed docking with the AutoDock Vina docking engine, and calculated the structure-truncated MM/PB(GB)SA energy and per-residue energy decomposition based on multiple poses. The receptor–ligand complex obtained from CB-Dock2 was used as the binding pocket reference, and then 10 pose numbers were selected. For pose rescoring, receptor forcefield ff99SB (with TIP3P water model) and ligand force field GAFF2 were selected, and the truncation radius setting was kept at the default value with all rescoring procedures. The hotspot predictions were carried out with an unaltered ff99SB forcefield and default truncation radius. To evaluate the docking poses, MM/PBSA and MM/GBSA (to yield an energy decomposition on a per-residue basis) were submitted separately. The results obtained were downloaded and used for further analyses.

### Molecular dynamics simulation

All molecular dynamics (MD) simulations were performed using the AMBER 16 package with the ff99SB and GAFF force fields for the receptors RpfB and FadD, and the ligand DSF^69^. The antechamber module of AmberTools was used to calculate the partial charges of the ligand using the semi-empirical AM1-BCC function according to the standard protocol^70^. The complexes were solvated with the TIP3P water model and neutralized by adding Na + ions using the tLEap input script from the AmberTools package. Long-range electrostatic interactions were modeled using the particle-mesh Ewald method^71^. The SHAKE algorithm^72^ was applied to constrain the length of covalent bonds, including the hydrogen atoms. Langevin thermostat was implemented to equilibrate the temperature of the system at 310 K. A 2.0-fs time step was used in all the MD setups. For the minimization and equilibration (NVT and NPT ensembles) phases, 100,000 steps and a 1-ns period were used, respectively. Finally, 100 ns classical MD simulations, with no constraints as NPT ensemble, were performed for each of the receptor–ligand complexes using molecular mechanics combined with the Poisson–Boltzmann (MM-PBSA) or generalized Born (MM-GBSA) method augmented with the hydrophobic solvent-accessible surface area term^73,74^. The MM-PBSA/GBSA solvation models were applied as a post-processing end-state method to calculate the free energies (ΔG_pbsa_ and ΔG_gbsa_).

## Supplementary Information

Supplementary Information 1.

Supplementary Information 2.

## Data Availability

The dataset GSE4143 analyzed during the current study is available at https://www.ncbi.nlm.nih.gov/geo/query/acc.cgi?acc=GSE4143.
