# Supplementary material for: DSF inactivator RpfB homologous FadD upregulated in Bradyrhizobium japonicum under iron limiting conditions
Source: Sci Rep. 2023 May 29;13:8701. doi: 10.1038/s41598-023-35487-9 (PMC10226988; doi:10.1038/s41598-023-35487-9)
Supplement: Supplementary file 1 — Supplementary Information 1. [file 41598_2023_35487_MOESM1_ESM.docx]

SUPPLEMENTAL SECTION


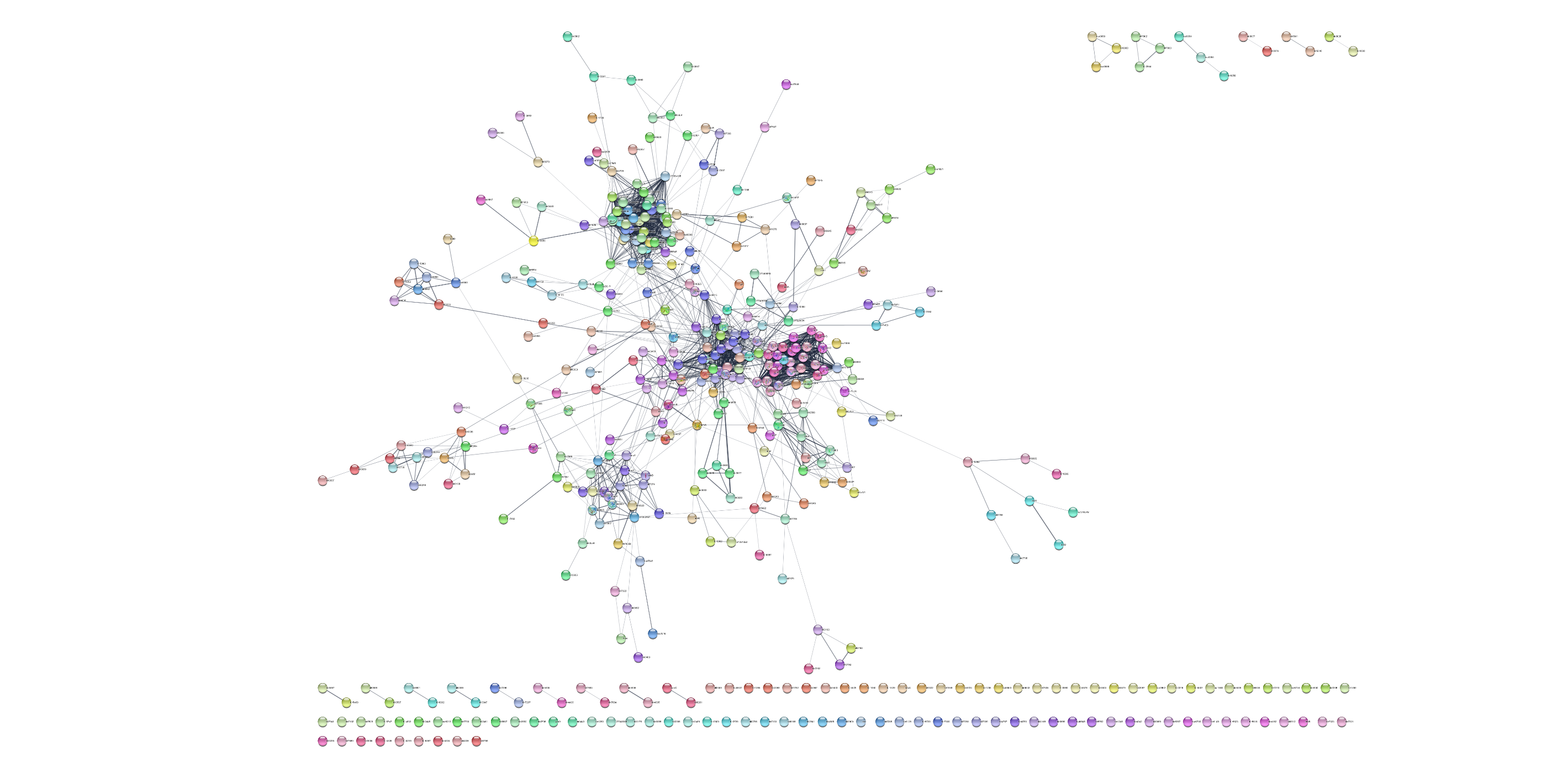
 Figure S1. Compete protein–protein interacting network of the differentially expressed genes between *Bradyrhizobium japonicum* grown in low or high iron conditions. (a) Interacting nodes in the FadD, quorum sensing, and ABC transporters clusters. (b) Flagellar assembly network. (c) Ribosome interacting network. (d) Root nodulation interacting nodes.

(d)

(c)

(b)

(a)

.

(a)


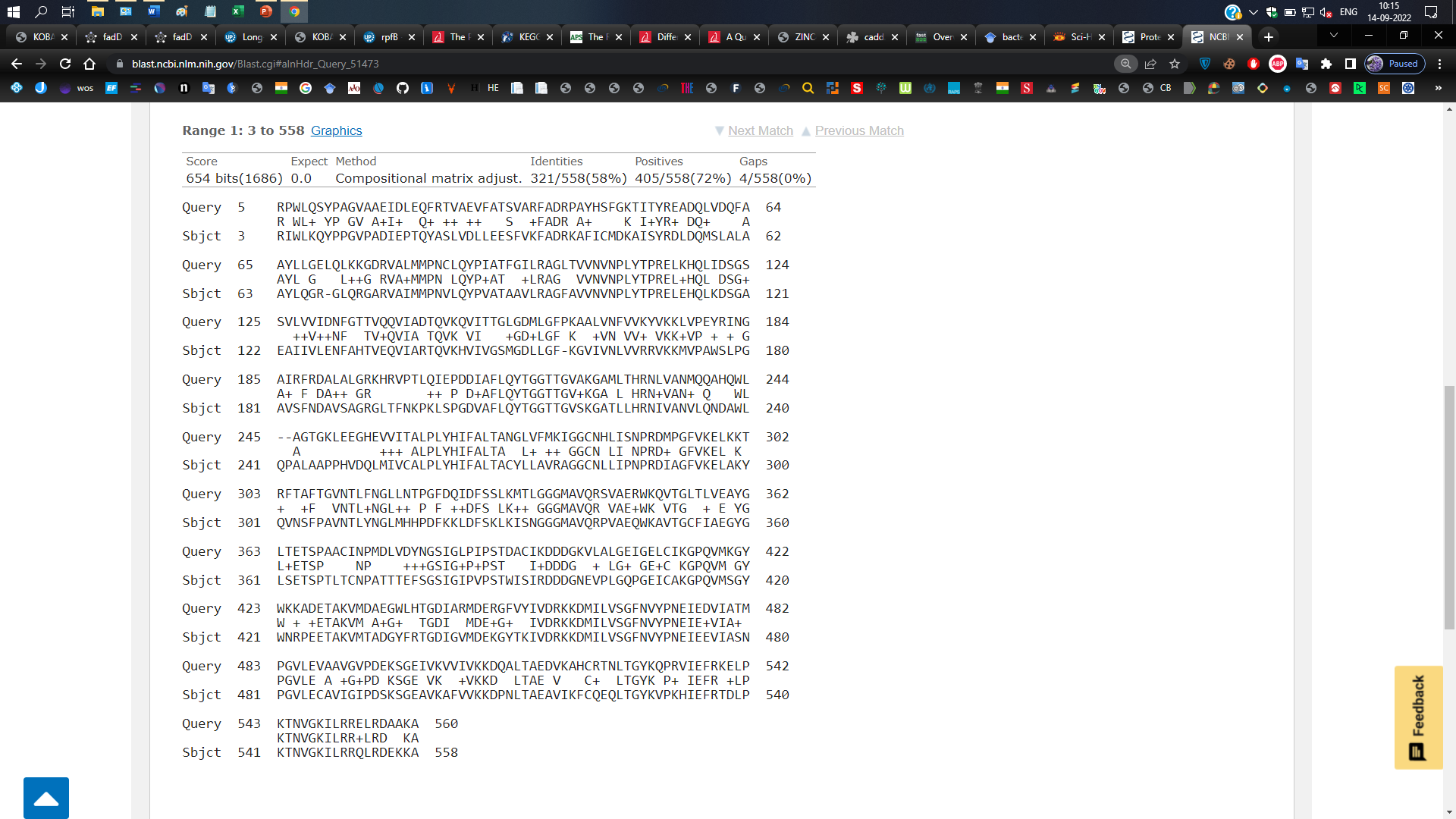


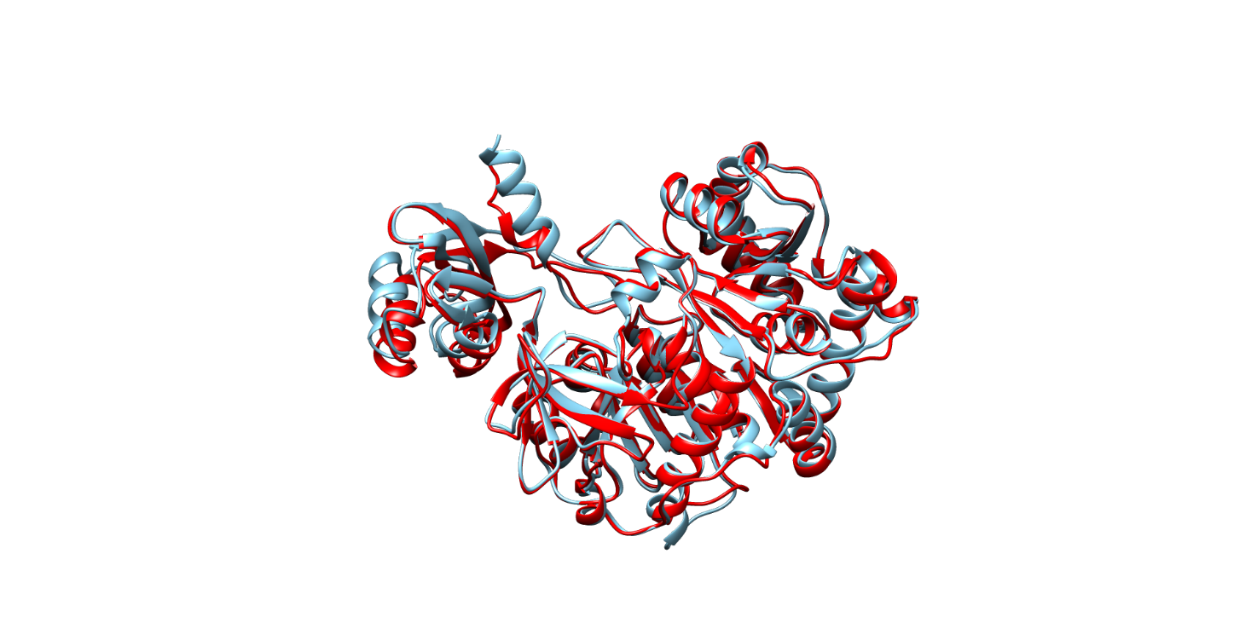

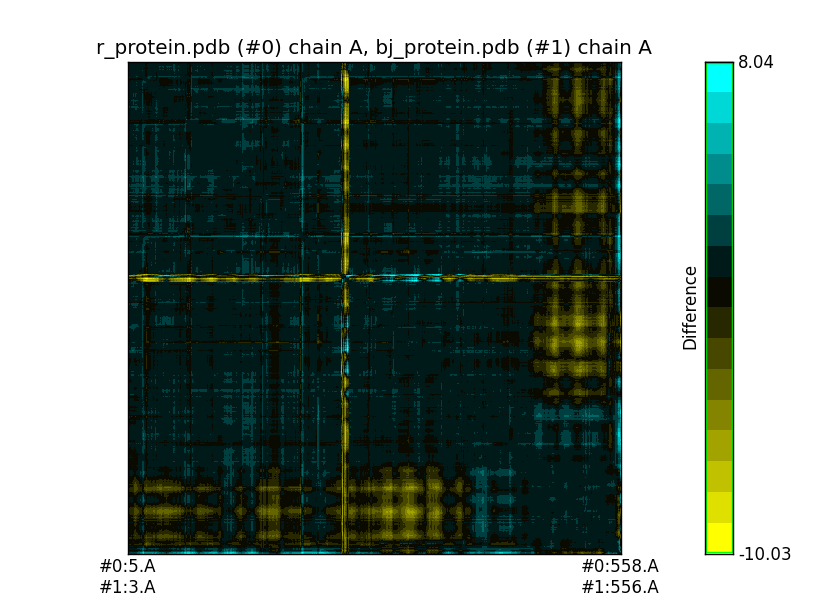


(b)

(c)

Figure S2. Comparative sequence and structure analyses between RpfB and FadD. (a) Alignment of the amino acid sequences of RpfB and FadD. The sequences were obtained from UniProt, and the sequence alignment was analyzed using the NCBI multiple sequence analysis user interfaces. RpfB, semi-transparent gold; FadD, semi-transparent blue. (b) AlphaFold predicted structures of RpfB (AF-Q8P9K5-F1) and FadD (AF-A0A0A3XRM6-F1) were compared using the UCSF Chimera. RpfB, red; FadD, cyan. (c) RRDistMap of RpfB and FadD.


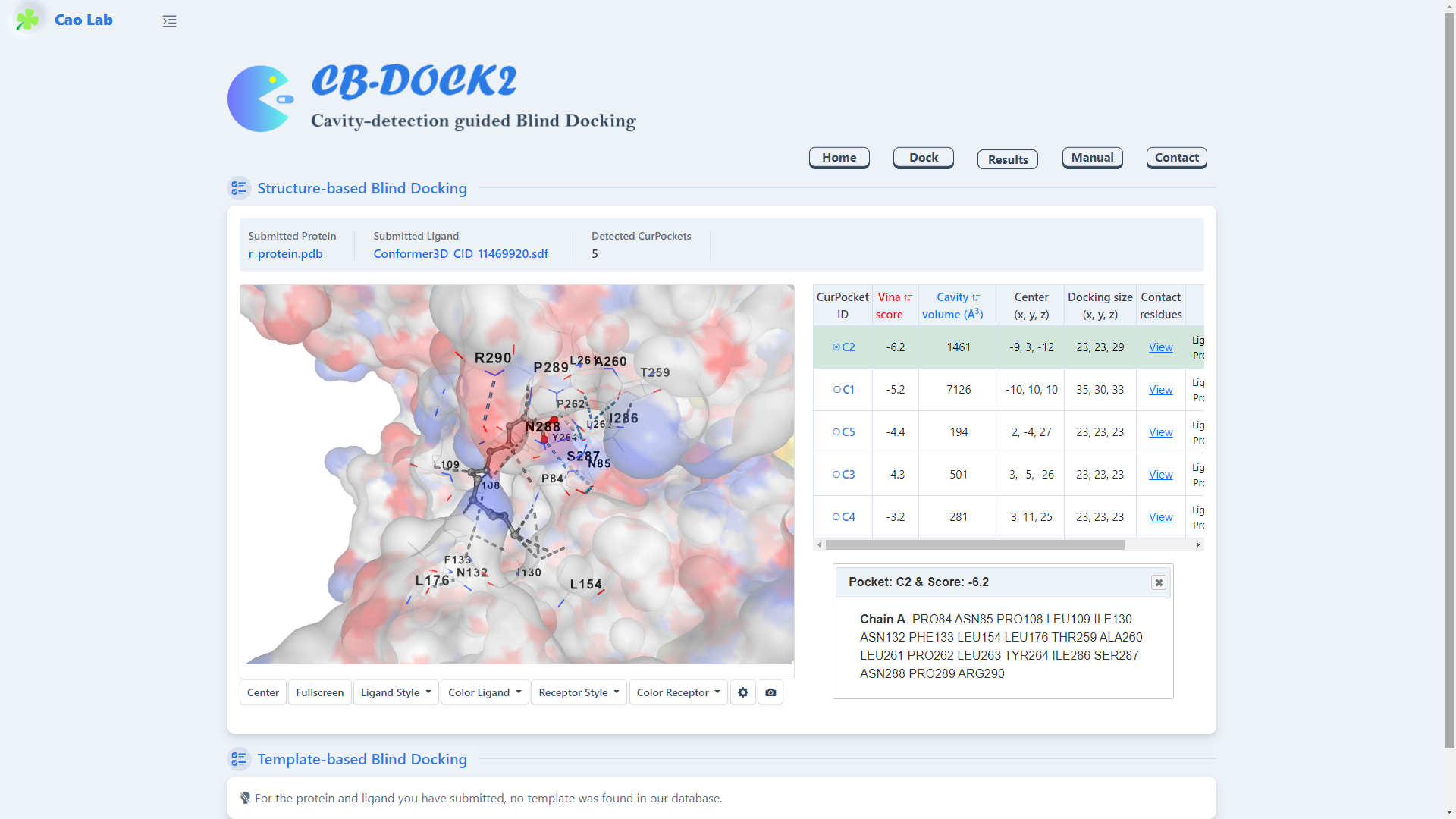

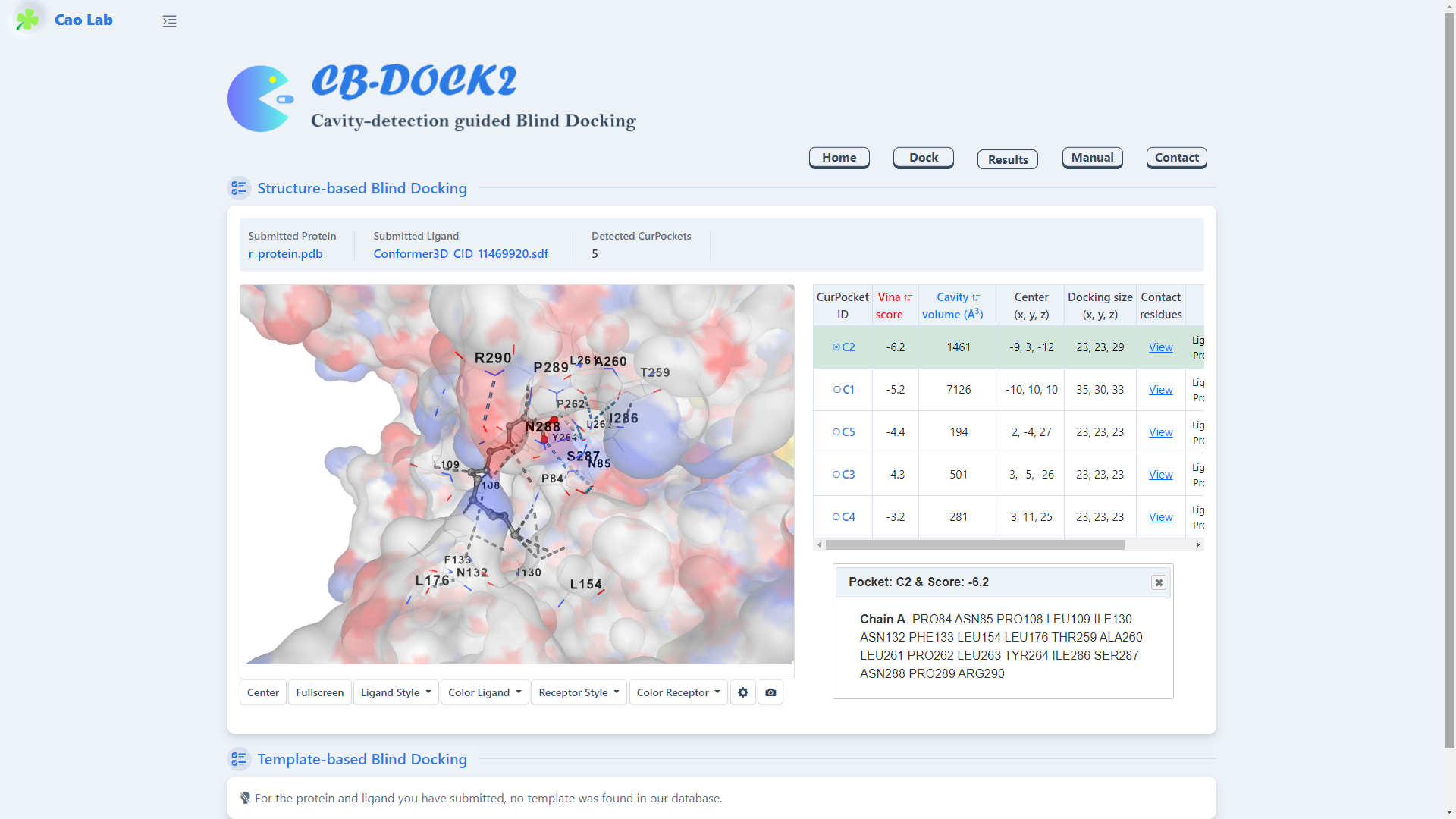

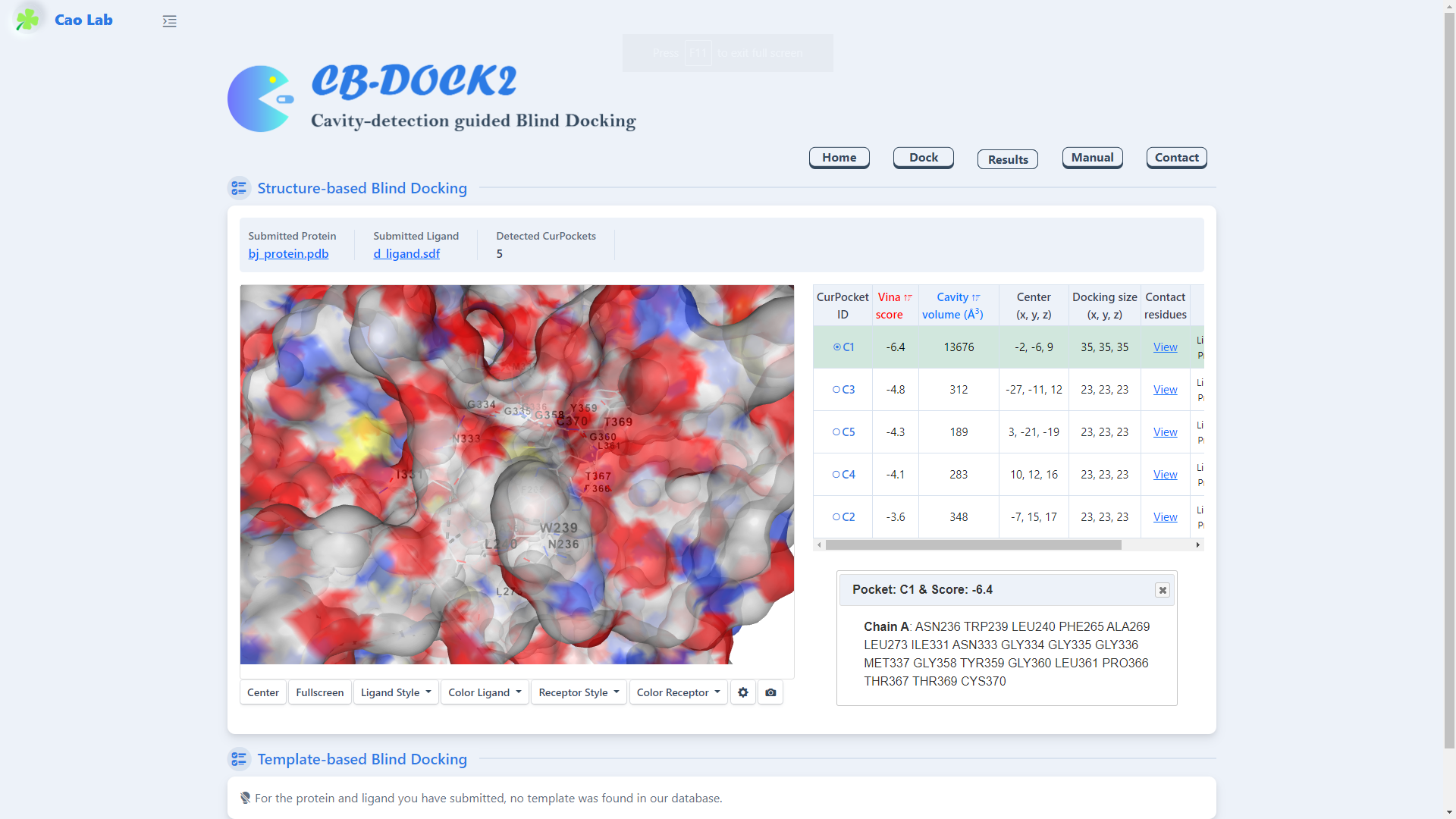


(b)

(a)


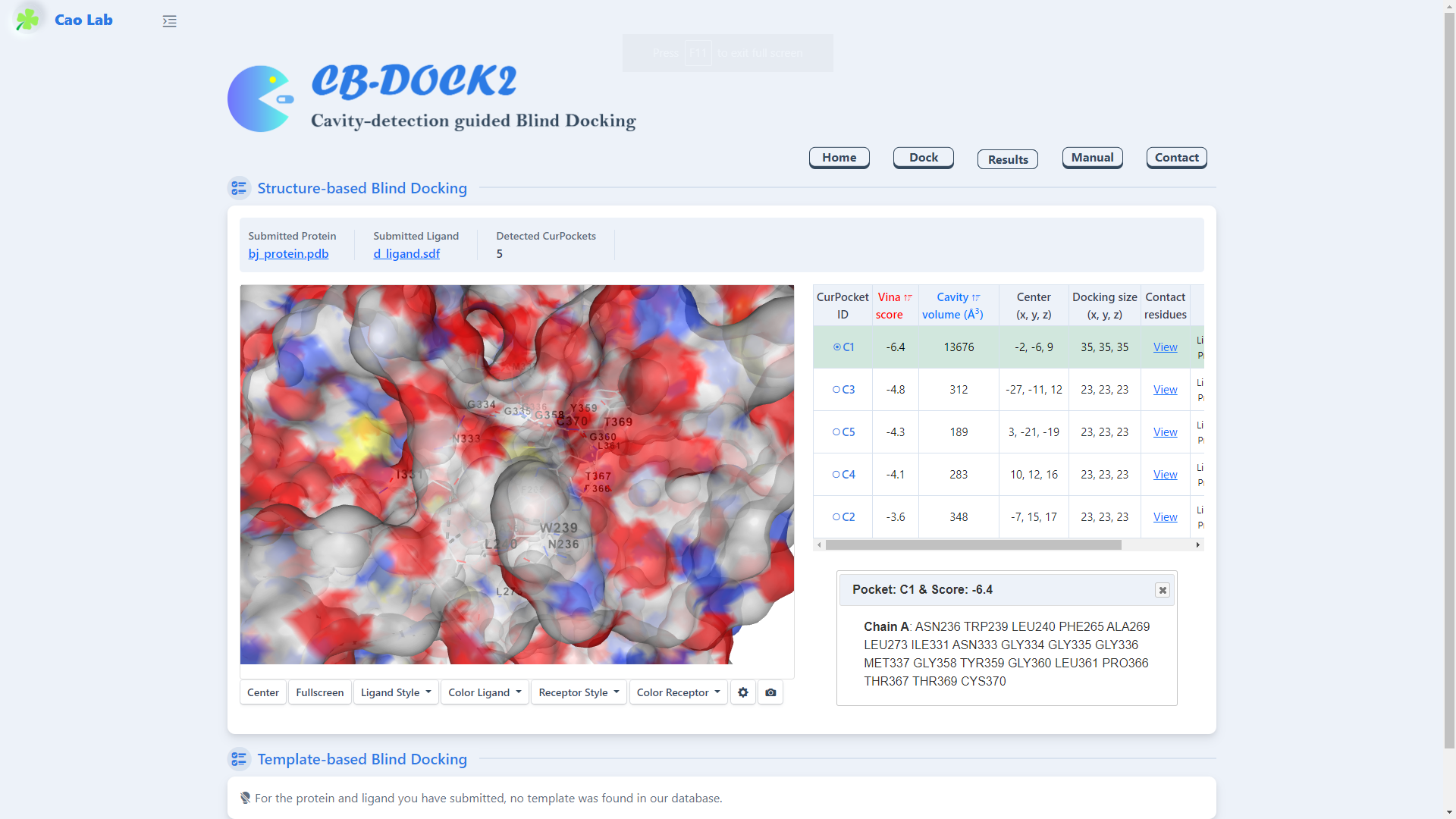


Figure S3. Contact residues identified by CB-Dock2 molecular docking. (a) RpfB–DSF and (b) FadD–DSF.


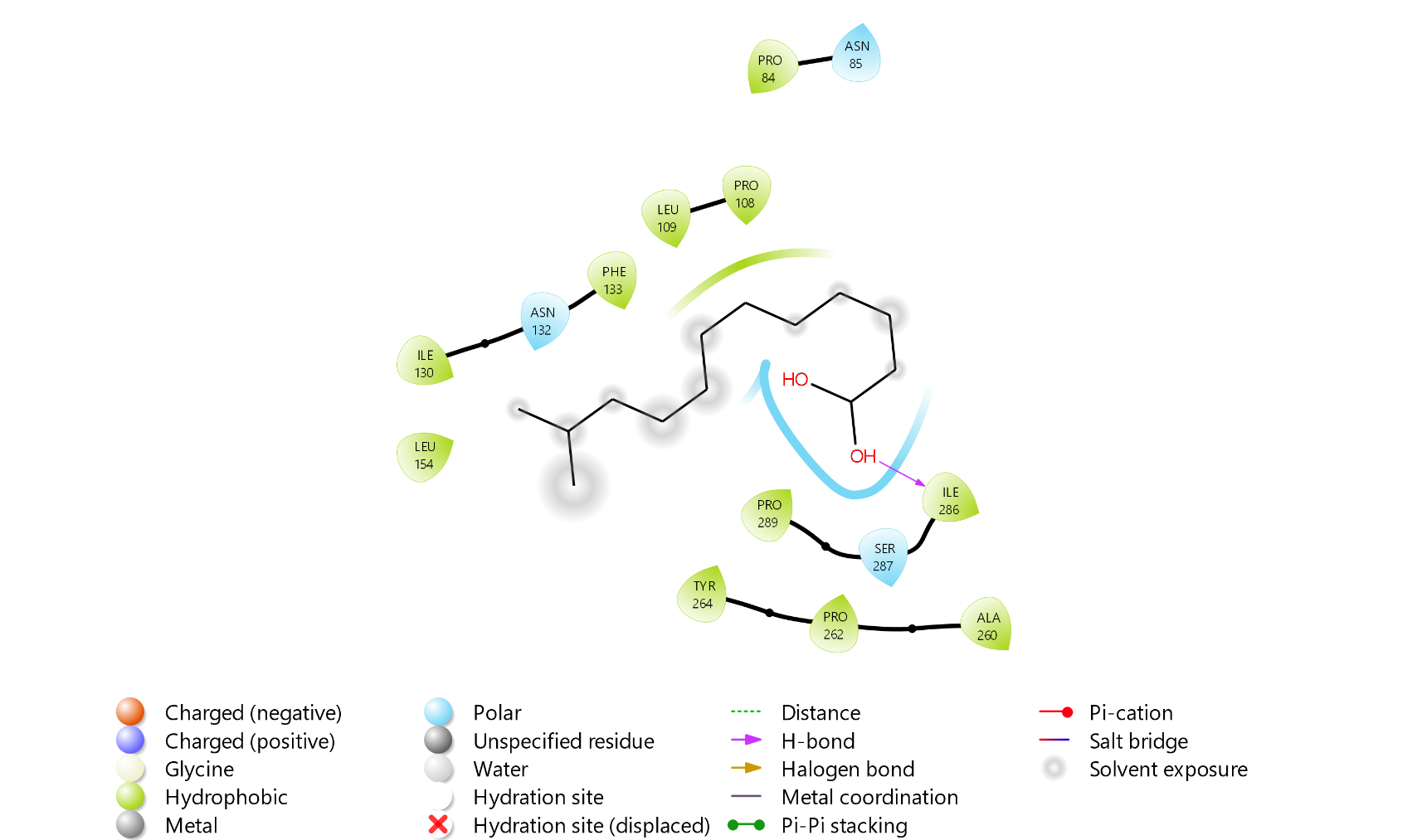

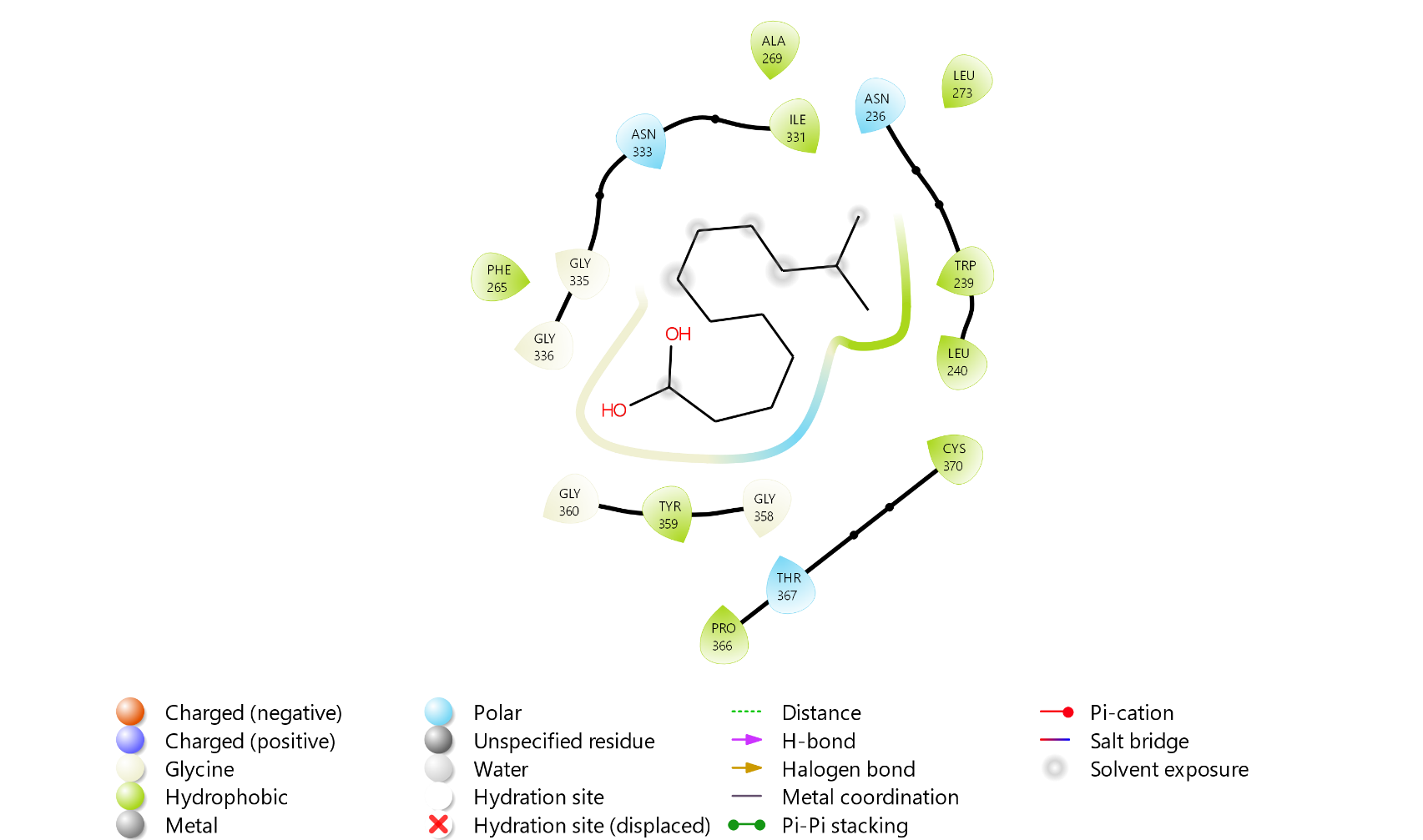


(b)

(a)


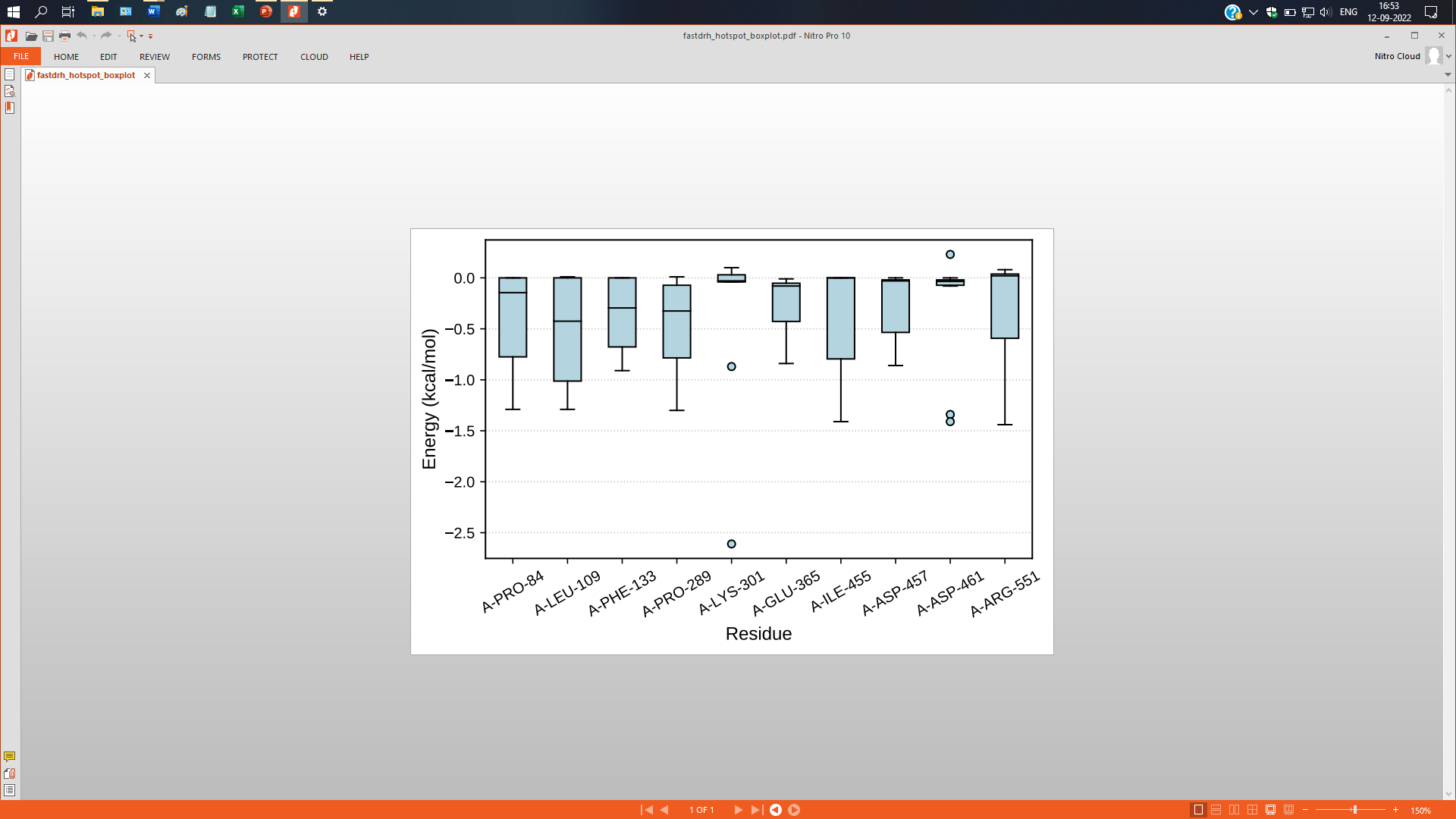

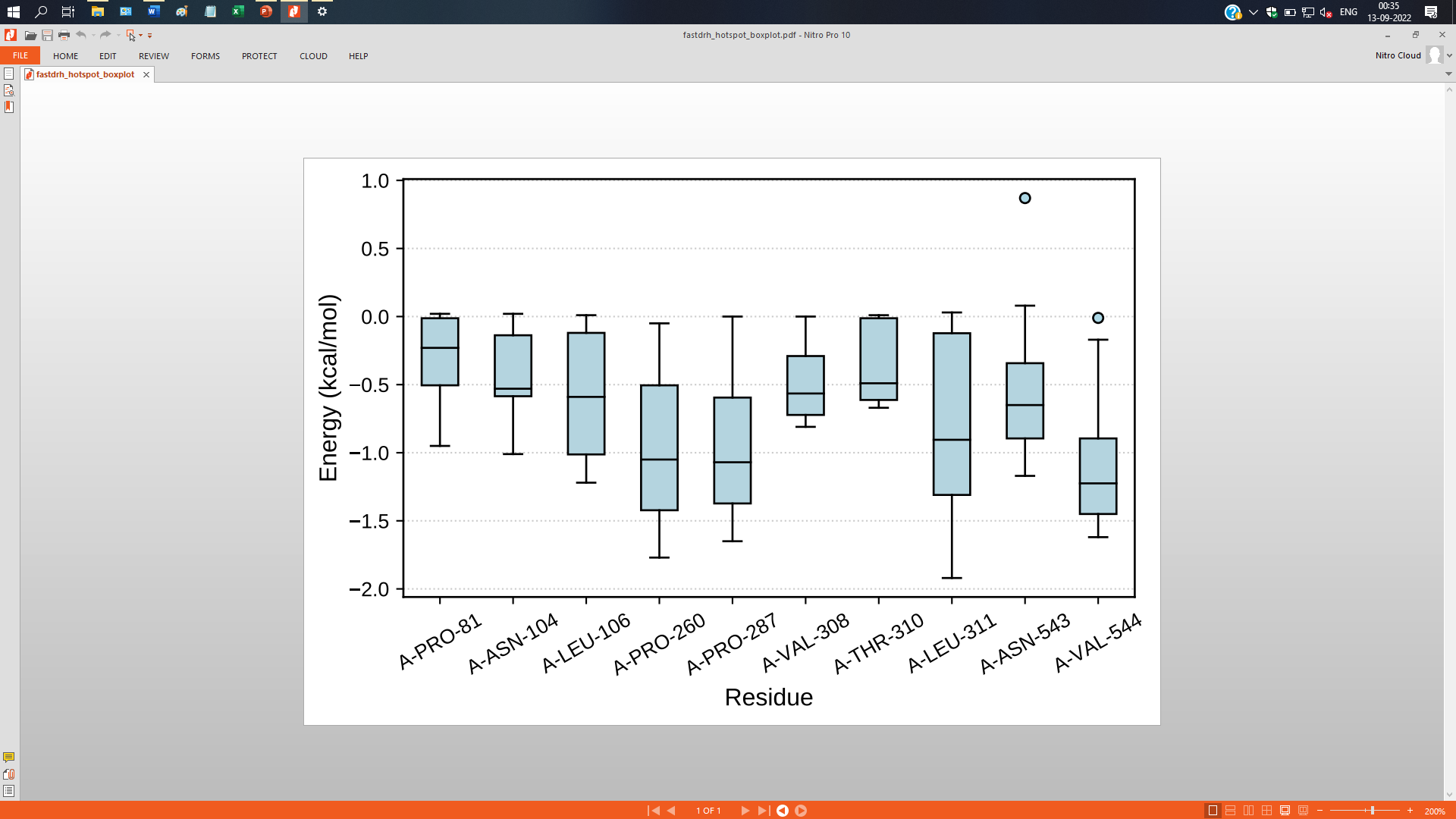


(e)

(c)

(d)


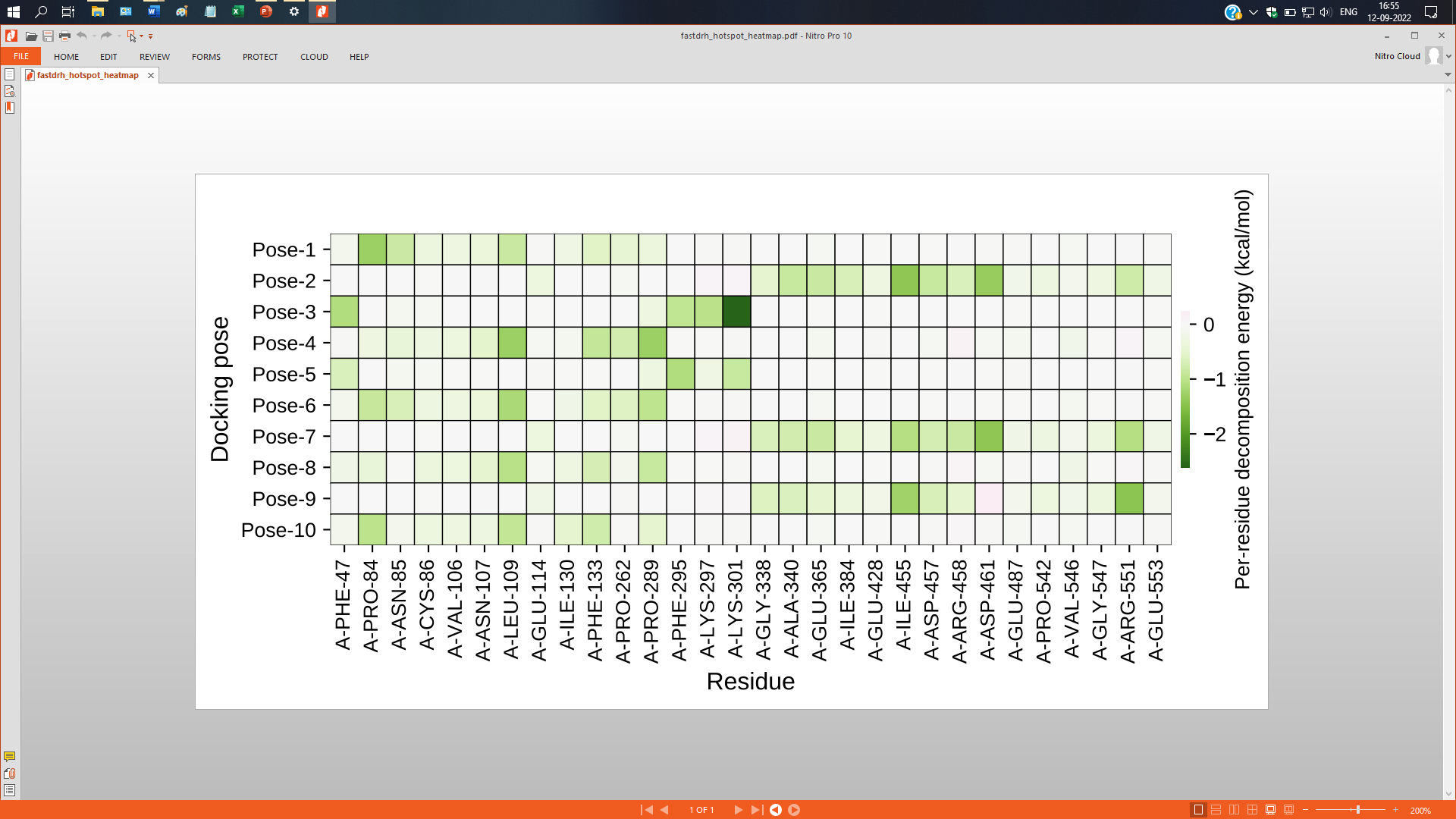


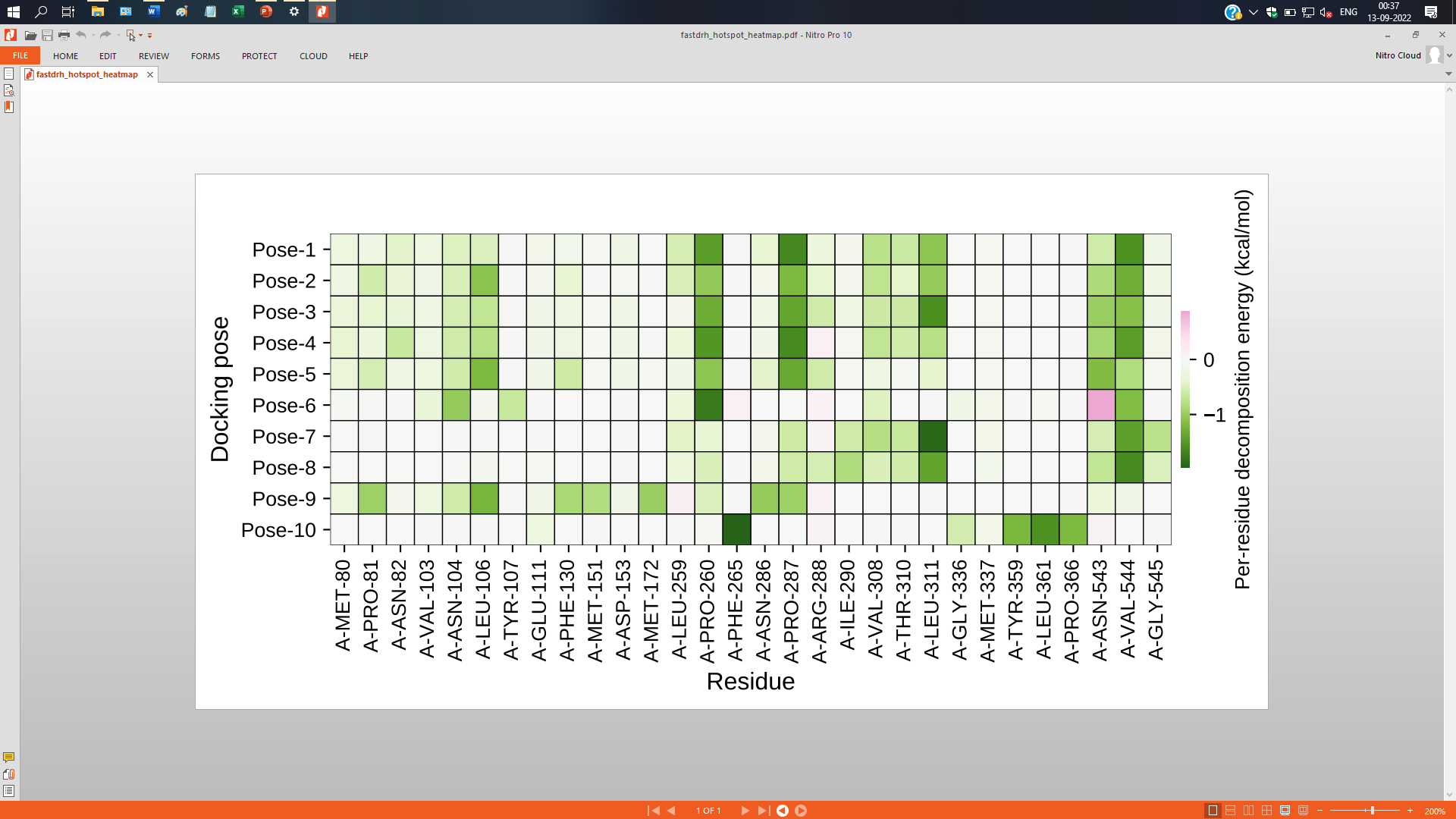


(f)

Figure S4. Molecular docking and rescoring. (a, b) Molecular interactions between DSF and RpfB of *Xcc* (a) and between DSF and FadD of *Bradyrhizobium japonicum* (b). (c, d) Binding energy per amino acid residue (MM-PBSA) of the top 10 potential hotspot residues between DSF and RpfB of *Xcc* (c) and between DSF and FadD of *B. japonicum* (d). (e, f) Heatmap of the top 30 residues of RpfB (e) and FadD–DSF (f).

| Table S2. KEGG pathways of differentially expressed genes of *Bradyrhizobium japonicum* between low and high iron conditions. | | | | |
| --- | --- | --- | --- | --- |
| KEGG Pathways | ID | Input number | Background number | Corrected P-Value |
| Flagellar assembly | bja02040 | 19 | 60 | 9.06e^-17^ |
| Ribosome | bja03010 | 16 | 58 | 1.60e^-13^ |
| Oxidative phosphorylation | bja00190 | 14 | 67 | 1.47e^-10^ |
| Metabolic pathways | bja01100 | 38 | 1190 | 5.37e^-05^ |
| Carbon fixation in photosynthetic organisms | bja00710 | 5 | 29 | 0.0011 |
| Sulfur relay system | bja04122 | 3 | 13 | 0.0114 |
| Porphyrin and chlorophyll metabolism | bja00860 | 4 | 40 | 0.0244 |
| Biosynthesis of secondary metabolites | bja01110 | 14 | 414 | 0.0244 |
| Beta-Lactam resistance | bja01501 | 3 | 21 | 0.0252 |
| Carbon metabolism | bja01200 | 8 | 176 | 0.0266 |
| Microbial metabolism in diverse environments | bja01120 | 14 | 445 | 0.0324 |
| Glyoxylate and dicarboxylate metabolism | bja00630 | 5 | 87 | 0.0432 |
|  | | | | |

| Table S3. Genes and associated pathways of differentially expressed genes of *Bradyrhizobium japonicum* between low and high iron conditions. | |
| --- | --- |
| KEGG Pathways | List of genes |
| Flagellar assembly | *fla ,fliF ,flhA ,flgB ,fliQ ,fliP ,fliR ,flgC ,fliE ,flgA ,fliN ,flgF ,flgE ,flgD ,flgK ,flgI ,flgH ,fliG ,flgL* |
| Ribosome | *rpsF ,rplB ,rplX ,rplW ,rpsL ,rpsK ,rplP ,rplC ,rplN ,rpsS ,rpsQ ,rplF ,rplD ,rplE ,rpmC ,rpmD* |
| Oxidative phosphorylation | *coxA ,nouD ,nuoB ,nuoC ,coxE ,nuoA ,nuoF ,nuoG ,nuoJ ,nuoI ,nuoN ,sdhD ,nuoL ,nuoM* |
| Metabolic pathways | *dapE ,pgsA ,moaE ,cbbA ,acnA ,fadD ,birA ,cbbT ,cbbP ,cbbS ,lipA ,murI ,gph ,nrdE ,dhlB ,fumC ,rbcL ,leuC ,sdhD ,nosZ ,nouD ,coxE ,pyrG ,coxA ,hemB ,hemA ,nuoB ,nuoC ,nuoA ,nuoF ,nuoG ,nuoJ ,glcF ,nuoI ,nuoN ,ispA ,nuoL ,nuoM* |
| Carbon fixation in photosynthetic organisms | *cbbT ,cbbA ,cbbP ,cbbS ,rbcL* |
| Sulfur relay system | *moaE ,moaD ,mnmA* |
| Porphyrin and chlorophyll metabolism | *hemB ,bfr ,hemA ,coxE* |
| Biosynthesis of secondary metabolites | *ispA ,gph ,leuC ,hemB ,hemA ,cbbA ,fumC ,rbcL ,cbbT ,glcF ,acnA ,sdhD ,cbbS ,coxE* |
| beta-Lactam resistance | *acrB ,ampC ,acrA* |
| Carbon metabolism | *cbbA ,fumC ,cbbP ,rbcL ,cbbT ,acnA ,sdhD ,cbbS* |
| Microbial metabolism in diverse environments | *dapE ,dhlB ,hemB ,nosZ ,cbbA ,hupL ,fumC ,rbcL ,cbbT ,glcF ,acnA ,cbbP ,cbbS ,sdhD* |
| Glyoxylate and dicarboxylate metabolism | *glcF ,gph ,rbcL ,cbbS ,acnA* |

| Table S4. Protein folding patterns and protein family domains shared by FadD of *Bradyrhizobium japonicum* and RpfB of *Xanthomonas campestris* pv. *campestris*. | | | |
| --- | --- | --- | --- |
| Types | Accession | FadD | RpfB |
| Domain | IPR000873  AMP-binding enzyme - PF00501 | 30 – 463 | 31 – 465 |
|  | IPR025110  AMP-binding_C - PF13193 | 472 – 546 | 474 – 548 |
| Homologous  superfamily | IPR042099  G3DSA:3.40.50.12780 | 13 – 458 | 14 – 460 |
|  | IPR045851  G3DSA:3.30.300.30 | 459 – 560 | 461 - 559 |
| Conserved Site | IPR020845  AMP_BINDING - PS00455 | 209 – 220 | 213 – 224 |
| Unintegrated | FC-FACS_FadD_like - cd05936  Putative AMP binding site  Acyl-activating enzyme (AAE) consensus motif  Putative CoA binding site  Putative active site | 25 – 553 | 27 – 555 |
|  | Acetyl-CoA synthetase-like - SSF56801 | 11 – 557 | 23 – 558 |
| Protein sequence, UniProt ID: A0A0A3XRM6 (FadD), Q8P9K5 (RpfB) | | | |
